# Functional Profiling in Paralympic Water Polo Using Deep Learning, Stereo Vision, and Phase-Based Kinematic Analysis: A Pilot Study

**DOI:** 10.3390/bioengineering13060707

**Published:** 2026-06-19

**Authors:** Andrea Zanela

**Affiliations:** Energy and Data Science Lab, ENEA “Casaccia” Research Centre, I00123 Rome, Italy; andrea.zanela@enea.it

**Keywords:** paralympic classification, paralympic water polo, functional assessment, pose estimation, stereo vision, markerless motion capture, phase-based analysis

## Abstract

Paralympic water polo requires classification systems that reflect sport-specific functional performance under ecologically valid conditions. This pilot study proposes a task-specific kinematic profiling framework for deriving objective, biomechanically interpretable descriptors of residual motor function. Five male national-level water polo athletes—three with eligible motor impairments and two able-bodied reference participants—performed standardized sport-specific tasks comprising upright floating, vertical propulsion, unilateral passing, non-contested shooting, and contested shooting under physical opposition. Stereoscopic video, OpenPose-based three-dimensional reconstruction, and phase-based analysis were used to extract features and composite indices of postural control, propulsion capacity, upper-limb residual function, and resistance to perturbation. Automatic ball-release detection matched manual frame-level verification in all 128 analyzed ball-related trials. Within the task-specific indices, where higher scores indicate greater functional burden, core values ranged from 0.05–0.15 for upright floating, 0.29–0.68 for combined arm-and-leg vertical propulsion, and 0.040–0.148 for contested shooting across the available subject–side combinations. The profiles showed task- and side-specific differences in stabilization, propulsion, and post-contact motor reorganization. The framework uses pose estimation as a quantitative measurement tool and treats visibility interruptions as functionally meaningful events rather than noise. It is not intended to replace official classification procedures, but to provide transparent and interpretable candidate descriptors for future evidence-based classification research in Paralympic water polo.

## 1. Introduction

The expansion of Paralympic sport has intensified the need for transparent, evidence-based, and sport-specific classification systems capable of ensuring fair competition. The primary aim of classification is to minimize the impact of impairment on competition outcomes so that success reflects training, skill, and tactical ability rather than severity of disability [1,2]. This principle has progressively shifted classification from predominantly medical models toward function-based systems that evaluate how eligible impairments affect sport-specific performance [3,4].

In Paralympic water polo, classification is particularly challenging because performance depends on the interaction of multiple functional domains, including postural control, buoyancy management, vertical propulsion, unilateral upper-limb coordination, and tolerance to physical contact. Unlike cyclic or highly constrained tasks, water polo actions are intermittent, multidirectional, and frequently perturbed by external forces. As a result, classification requires assessment tools capable of capturing how impairments affect performance under ecologically valid, game-relevant conditions rather than in isolated or overly simplified tests [2,5].

Current classification procedures in Paralympic sport generally combine physical assessment, technical evaluation, and observation during competition. Although clinical examination protocols are relatively standardized, the technical and observational components may still rely partly on qualitative judgement, particularly in complex and contact-rich environments. In these settings, subtle compensatory strategies, inter-limb asymmetries, and reduced robustness under perturbation may be difficult to quantify objectively, potentially affecting consistency and transparency in classification decisions [2,5].

Recent advances in computer vision and markerless motion analysis provide new opportunities for objective functional assessment in sport and rehabilitation. Deep learning-based pose estimation frameworks such as OpenPose enable extraction of human kinematics from video data without the need for wearable sensors or reflective markers [6]. When combined with stereoscopic vision, these approaches support three-dimensional motion reconstruction in real-world environments where conventional motion-capture systems may be impractical [7,8,9]. Previous studies have also shown that video-based pose estimation and quantitative kinematic analysis can provide biomechanically meaningful descriptors of motor performance in both healthy and clinical populations, supporting their potential use for quantitative functional assessment [10,11].

Recent methodological and validation studies have developed multi-camera OpenPose-based workflows and evaluated markerless kinematic measurements against marker-based motion capture or manually digitized reference data across walking, running, cycling, jumping, throwing, and real-world athletics. These studies support the feasibility of markerless kinematic analysis while also showing that accuracy depends on the movement, camera configuration, processing pipeline, and biomechanical variable considered [12,13,14,15,16]. Previous work by the authors has applied AI-based video analysis, alone or in combination with inertial sensing, to sport-specific performance assessment and technically complex actions, including springboard diving [17,18,19]. Broader reviews and additional applications in underwater swimming and field-based sport further underline both the potential and the context-dependent limitations of markerless motion capture [20,21,22,23].

However, most existing vision-based approaches assume continuous and fully observable kinematics. This assumption is rarely met in aquatic and contact-rich sports. In water polo, partial or complete occlusion of body segments due to submersion is intrinsic to task execution rather than a simple measurement artifact. Methods based on continuous trajectory reconstruction may therefore overlook functionally relevant aspects of performance, especially in contested situations where submersion and re-emergence are part of the motor behavior being evaluated.

To address these limitations, the primary aim of this pilot study was to assess the feasibility of a task-specific markerless kinematic profiling framework for quantifying residual motor function during representative Paralympic water polo actions. Specifically, the study aimed to: (i) derive three-dimensional, biomechanically interpretable descriptors of postural control, vertical propulsion, unilateral upper-limb function, and resistance to physical perturbation; (ii) define task-specific phase and event segmentation strategies suited to aquatic and contact-rich conditions, including release-centered analysis and the explicit treatment of submersion-related visibility loss; and (iii) aggregate the resulting descriptors into compact functional indices supporting side-, task-, and subject-specific profiling.

The main methodological contribution is a unified phase-based framework that combines stereoscopic three-dimensional pose reconstruction, automatic ball-release anchoring, task-specific descriptor extraction, and composite functional profiling, while explicitly preserving visibility interruptions and submersion-related discontinuities as potentially meaningful components of task execution.

A secondary exploratory aim was to examine whether the proposed descriptors and indices captured distinct patterns of functional performance across tasks, body sides, and individual participants. The study was not designed to validate Sport Class assignment or to establish classification thresholds. Rather, it provides a transparent methodological framework and a set of candidate sport-specific descriptors to be evaluated in future studies involving larger cohorts, repeated-session reliability, and comparison with expert classification assessments. The remainder of the paper describes the acquisition and analysis framework, presents the resulting functional profiles, and discusses their methodological relevance and current limitations.

## 2. Materials and Methods

This section describes the study design, participant cohort, stereoscopic acquisition system, pose-estimation and signal-processing procedures, task-specific segmentation strategy, feature extraction, and formulation of the functional indices. Detailed operational criteria, processing parameters, and descriptor definitions are provided in the Supplementary Materials. A high-level overview of the complete acquisition and processing workflow, from stereoscopic video recording to task-specific functional profiling, is provided in Supplementary Figure S1.

### 2.1. Study Design and Functional Assessment Framework

This pilot study was designed to develop a task-specific kinematic profiling framework for exploratory functional characterization in Paralympic water polo, with the aim of identifying objective and biomechanically interpretable descriptors potentially relevant to future classification-oriented evaluation. In the present study, these descriptors should be regarded as candidate functional markers rather than validated classification indicators. The proposed approach does not aim to automate Sport Class assignment, but rather to extract objective and biomechanically interpretable descriptors of residual motor function during ecologically valid gameplay actions, in accordance with established classification principles [1,24].

Because classification in Paralympic sport concerns the impact of eligible impairments on sport-specific performance, the experimental protocol was structured around standardized sport-specific tasks representing fundamental water polo movement patterns. Each task was conceived as a functional probe targeting a specific motor domain rather than as a generic performance test.

The protocol included the following tasks:

• upright floating
• explosive vertical propulsion from upright posture performed with:
  - combined arms and legs contribution
  - legs only (arms crossed on the chest)
• unilateral passing (with the right and left arm)
• non-contested shooting (with the right and left arm)
• contested shooting under physical opposition (right and left arm)

Together, these tasks were designed to probe distinct but complementary functional domains relevant to gameplay, including postural control and buoyancy management, vertical propulsion capacity, unilateral upper-limb propulsion and proximal–distal coordination, and robustness to perturbation with post-contact motor reorganization.

For dynamic upper-limb tasks, right and left arms were analyzed independently to capture possible asymmetries in residual function and compensatory strategy.

All tasks were analyzed using a phase-based segmentation approach aligned with their functional structure. Actions involving physical opposition were decomposed into distinct phases reflecting pre-perturbation control, perturbation/submersion, and post-contact reorganization. This approach explicitly accounts for the intrinsic discontinuities of aquatic and contact-rich environments, where temporary loss of KeyPoint visibility is treated as a functionally meaningful event rather than as measurement noise [8].

### 2.2. Participants

Data were collected at the swimming pool of the Centro di Preparazione Olimpica “Giulio Onesti” (CONI, Rome, Italy). Five male participants were enrolled in this pilot study after providing written informed consent.

Three participants presented permanent motor impairments eligible under the classification framework adopted for the study, whereas two able-bodied water polo athletes were included as reference participants. Individual anthropometric characteristics, general impairment information, the main affected body region or side, and official Sport Class, when available, are reported in Table 1.

The athletes with eligible impairments presented different functional profiles and were included to explore the ability of the proposed framework to describe heterogeneous, subject-specific motor strategies. The able-bodied participants were included solely as qualitative reference profiles and were not intended to constitute a normative control group.

All participants were recruited from national-level water polo programs and were familiar with the execution of the experimental tasks. Given the pilot and feasibility-oriented design, no inferential comparison between participant groups was planned. The small sample size therefore limits generalizability and does not support the definition of classification thresholds or population-level discriminability.

The effective number of acquired, retained, and excluded trials for each subject, task, and side is reported in Supplementary Table S1.

### 2.3. Data Acquisition System

Kinematic data were acquired using a stereoscopic video system based on a ZED2 camera (Stereolabs, San Francisco, CA, USA) [25]. The device integrates two horizontally aligned image sensors with a fixed baseline of 12 cm, enabling depth estimation through stereo triangulation.

Video sequences were recorded at 60 frames per second with a total resolution of 2560 × 720 pixels (1280 × 720 per sensor). This stereoscopic configuration allowed three-dimensional reconstruction of body KeyPoints from synchronized two-dimensional detections.

Stereo reconstruction was performed using camera parameters obtained through the manufacturer-provided recalibration procedure. All 3D trajectories were retained in the native ZED reference frame, without additional coordinate transformations. The default ZED coordinate system is right-handed, with X to the right, Y downward, and Z forward from the camera.

All motor tasks were recorded with fixed camera placement and orientation to ensure geometric consistency across participants and trials.

### 2.4. Pose Estimation and KeyPoint Selection

Human pose estimation was performed using the OpenPose software package (v1.5.1; Carnegie Mellon University, Pittsburgh, PA, USA) [6], a widely used open-source framework for multi-person 2D pose estimation. OpenPose follows a bottom-up approach and uses a feed-forward multi-stage convolutional neural network (CNN) to estimate 2D confidence maps for body-part locations together with 2D vector fields, known as Part Affinity Fields (PAFs), that encode the association between body parts.

**Figure 1:**
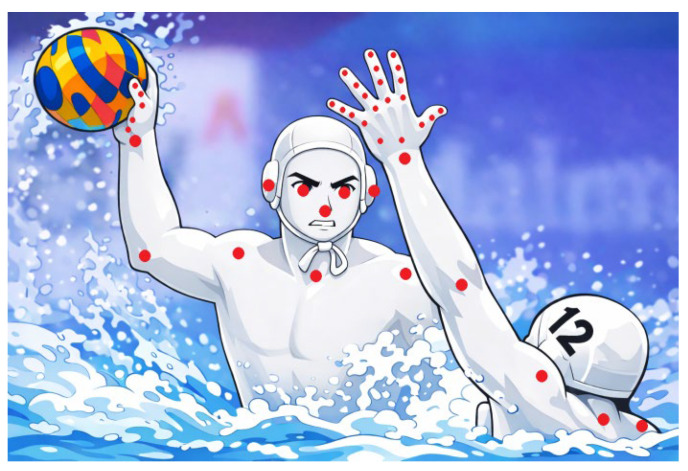
OpenPose KeyPoints considered in this study. The figure illustrates the anatomical landmarks detected by the BODY-25 and HAND-21 model in a representative water polo scenario.

In the present study, the BODY-25 and HAND-21 models were both computed throughout the acquisition pipeline. Because of the visibility constraints intrinsic to aquatic conditions, however, the availability and reliability of the detected KeyPoints depended on the actual observability of the corresponding anatomical landmarks in each frame. An overview of the anatomical KeyPoints considered in this study is provided in Figure 1.

For each detected KeyPoint, two-dimensional image coordinates and confidence scores were obtained independently from the left and right camera views. Three-dimensional coordinates were then reconstructed by stereo triangulation using the calibrated stereoscopic system described in Section 2.3.

Because visibility is inherently limited in aquatic environments, KeyPoints were selected separately for each task and functional phase. Only landmarks that met predefined thresholds for visibility, detection confidence, and temporal continuity were retained for quantitative analysis. The corresponding thresholds are reported in Supplementary Table S2. Lower-limb KeyPoints were generally excluded because they were systematically occluded by the water surface. When they were visible only intermittently, they were treated as unavailable rather than interpolated, thereby preserving the physical interpretability of the data.

This task-specific selection strategy enabled robust kinematic analysis under partial observability. Importantly, visibility interruptions were not treated as noise, but as an intrinsic component of task execution, particularly in contact-rich conditions where submersion may itself carry functional meaning.

**Figure 2:**
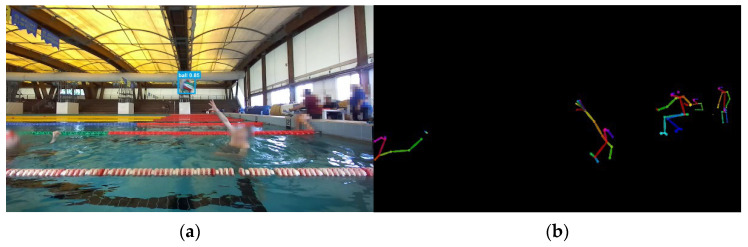
Example frame illustrating the vision-based analysis pipeline for ball-related tasks. (**a**) RGB view of a representative contested-shooting scene, showing YOLO-based ball detection with bounding box and confidence score; (**b**) corresponding multi-person OpenPose reconstruction in the same frame. Whole-body KeyPoints are shown for all detected persons in the scene. For ball-related tasks, ball localization and the spatial relationship between the detected ball and the active hand were used to identify the release frame, which served as the temporal anchor for release-centered feature extraction.

For ball-related tasks, human KeyPoint analysis was complemented by frame-wise ball detection. The ball was localized using a YOLO-based detector [26], and the release event was identified automatically by combining ball localization with the spatial relationship between the ball and the active hand, see Figure 2.

In practice, release timing was defined as the frame at which the ball was no longer in contact with the active hand and started an independent trajectory, as supported by its frame-to-frame relationship with the distal upper-limb KeyPoints. This automatically detected release event was then used as the central temporal reference for release-centered feature extraction in passing and shooting tasks.

To verify the reliability of this procedure, the automatically detected release frame was compared with manual frame-level annotation in all analyzed ball-related trials (n = 128), showing exact agreement in all cases.

### 2.5. Signal Processing

Raw KeyPoint trajectories contain high-frequency fluctuations arising from localization noise, water-surface perturbations, and non-functional micro-movements. To isolate physiologically meaningful dynamics, all coordinate trajectories were temporally filtered using a fourth-order low-pass Butterworth filter applied independently to each spatial component [27].

Filtering was performed with a zero-phase implementation to avoid temporal distortion of kinematic events and preserve the timing of peaks, onsets, and coordination-related measures.

Given the acquisition rate of 60 Hz, cut-off frequencies were selected to attenuate tracking noise while preserving task-relevant movement dynamics. For quasi-static conditions such as upright floating, a cut-off frequency of 3 Hz was used to capture dominant postural oscillations. Task-specific cut-off frequencies were selected according to movement dynamics. The exact cut-off values used for each task are summarized in Supplementary Table S3.

All kinematic features were computed from filtered trajectories only. No interpolation or smoothing was applied to compensate for missing KeyPoints, in order to preserve the functional meaning of tracking interruptions and avoid introducing artificial motion patterns.

When the minimum validity requirements were not satisfied, only the affected descriptor was treated as missing, whereas the remaining valid descriptors from the same trial were retained. The corresponding validity and exclusion rules are reported in Supplementary Table S2.

### 2.6. Phase-Based Task Segmentation

All motor tasks were segmented into functionally meaningful phases or analysis windows prior to feature extraction. Rather than relying on fixed trial durations or assuming continuous movement availability, segmentation was defined according to task-specific kinematic structure, visibility conditions, and, for ball-related actions, the timing of ball release.

For tasks not centered on ball release, segmentation was based on intrinsic movement events. In upright floating, analysis focused on short stable pre-propulsion intervals selected to isolate postural control under minimal voluntary movement. In explosive vertical propulsion, feature extraction was performed within event-centered propulsion windows aligned to the onset of upward motion and spanning pre-thrust, thrust, and post-thrust stabilization.

For ball-related tasks performed without external physical opposition, namely passing and non-contested shooting, feature extraction was organized within a release-centered framework. In these tasks, the automatically detected ball release served as the central temporal anchor, and descriptors were computed in fixed windows referenced to this event. When applicable, three temporal regions were considered: a pre-release window, a release-centered window, and a post-release or recovery window. This strategy reduced dependence on variable preparation duration and follow-through length while improving biomechanical interpretability and comparability across trials and subjects.

In contrast, tasks involving physical interaction required a dedicated phase-based representation reflecting the intrinsic discontinuities of movement. In contested shooting, three functional phases were defined:

• Pre-contact phase: upright control prior to physical contact, characterized by stable KeyPoint visibility
• Contact/submersion phase: interval of physical perturbation, often associated with partial or complete loss of visibility due to submersion
• Post-contact phase: re-emergence and motor reorganization leading to a shooting attempt

Phase boundaries were identified from the temporal continuity and visibility of head and trunk KeyPoints, primarily the nose and neck, and when available the shoulder midpoint.

The operational definitions adopted for phase boundaries and event anchoring are summarized in Supplementary Table S2.

The onset of the contact phase was defined as the last valid frame preceding sustained upper-body visibility loss, whereas re-emergence was defined as the first frame with restored upper-body visibility. When a valid post-contact release was available, this event was additionally used to refine the characterization of post-contact execution and to support comparison with the non-contested shooting condition.

Feature extraction was performed only within temporally contiguous segments meeting minimum visibility and continuity criteria. Frames affected by occlusion or submersion were excluded without interpolation. Segments not satisfying these requirements did not contribute to the corresponding descriptor, while the remaining valid descriptors from the same trial were preserved according to the validity rules reported in Supplementary Table S2.

This segmentation strategy aligns kinematic analysis with the functional organization of each task and explicitly treats tracking interruptions, submersion, and post-contact recovery as functionally meaningful components of performance rather than as measurement noise.

### 2.7. Task-Specific Feature Extraction

For each task, kinematic features were defined to capture biomechanically interpretable functional constructs rather than absolute positional metrics. Feature selection was task-specific and aimed to quantify distinct aspects of motor performance, including postural control, propulsion capacity, upper-limb coordination, compensatory whole-body involvement, and robustness to perturbation.

To facilitate interpretation and maintain consistency across tasks, features were organized into hierarchical functional tiers.

Tier 1 included robust event- or outcome-based variables, such as temporal measures, completion flags, and global performance indicators.

Tier 2 captured movement organization and reorganization dynamics, including variability, stabilization, and coordination-related descriptors.

Tier 3 described detailed task-specific kinematic characteristics, such as peak velocities, timing of propulsion, intersegmental coordination, and relative contribution of body segments.

When applicable, a fourth level of descriptors was introduced.

Tier 4 included derived integrated descriptors combining information across multiple features or phases to provide compact representations of higher-level functional constructs, such as recovery efficiency or compensatory burden.

For dynamic upper-limb tasks, wrist and elbow kinematics were analyzed relative to a torso reference, defined by the neck or shoulder midpoint, so that propulsion-related measures reflected arm-driven motion rather than whole-body translation.

Features that could not be reliably computed because of incomplete tracking or aborted actions were retained as missing values and excluded from subsequent aggregation without imputation, thereby preserving the functional meaning of incomplete or interrupted performance.

This tiered structure was applied consistently across all tasks, while the specific functional meaning of each tier was adapted to the biomechanical demands of the task.

### 2.8. Task-Specific Functional Indices

To provide compact and interpretable summaries of performance across tasks, the extracted features were aggregated into task-specific functional indices reflecting distinct motor constructs:

• Static Floating Index (SFI)
• Vertical Propulsion Index (VPI)
• Passing Arm Residual Index (PARI_R, PARI_L)
• Shooting Arm Residual Index (SARI_R, SARI_L)
• Contested Shooting Resistance Index (CSRI_R, CSRI_L)
• For the vertical propulsion task, two condition-specific indices were defined:
  - VPI_LA: explosive vertical propulsion using combined lower- and upper-limb contribution (legs + arms)
  - VPI_L: explosive vertical propulsion using lower-limb contribution only (legs only condition)

This distinction enables evaluation of propulsion performance under different mechanical constraints and supports interpretation of segmental contribution and compensatory strategy.

Feature values were aligned in physiological directionality so that higher values consistently reflect poorer residual functional performance. To preserve comparability across subjects, descriptors were scaled using fixed impairment-oriented rules defined at the feature level. Depending on the nature of the descriptor, scaling was implemented through bounded reference intervals or threshold-based bounded transformations. This approach preserves the interpretation of absolute performance differences while avoiding dependence on subject-specific distributions across repeated trials.

Indices were computed by aggregating available scaled descriptors only, automatically excluding missing values without imputation. More formally, each composite index was computed as shown in Equation (1):

(1) $$I=\frac{\sum_{i\in V}{w_{i}s}_{i}}{\sum_{i\in V}{w_{i}}_{i}}$$

where $s_{i}$ is the scaled score of descriptor i, $w_{i}$ is its assigned weight, and V is the set of valid descriptors available for the corresponding trial. The general scaling logic and descriptor-to-index composition adopted in the present study are summarized in Supplementary Table S4. This approach preserves interpretability in the presence of incomplete tracking, partially observed actions, or interrupted task execution.

The proposed indices are not intended to replace official Sport Class assignment. Rather, they provide structured and interpretable summaries of task-specific residual function. At the individual level, they support the identification of functional asymmetries, compensatory strategies, and domain-specific limitations; at the group level, they support structured comparison of task-specific functional profiles across subjects. The overall organization of tasks, functional domains, phase structure, feature tiers, and derived indices adopted in the proposed framework is summarized in Table 2.

### 2.9. Upright Floating

Maintaining a stable upright floating posture is a fundamental requirement in water polo, as it enables players to maintain body elevation above the water and to generate the vertical support needed for upper-limb actions [28,29].

Unlike terrestrial conditions, where stability benefits from ground support, athletes in water must continuously regulate body orientation in an inherently unstable environment.

From a functional perspective, upright floating reflects the ability to maintain vertical alignment and limit unnecessary oscillations while preserving readiness for action. This task therefore provides a direct probe of postural control under sport-specific aquatic constraints.

In the present protocol, upright floating was not assessed through dedicated prolonged trials but was extracted from short pre-propulsion segments embedded within dynamic tasks. This approach preserves ecological validity while isolating short periods of postural steadiness immediately preceding voluntary movement.

#### 2.9.1. Feature Extraction

Upright floating was analyzed using three-dimensional trajectories of upper-body KeyPoints that remained consistently visible above the water surface. The selected landmarks included the nose (BO00), used as a proxy for head stability; the neck (BO01), representing trunk alignment; and the left and right shoulders (BO05, BO02), enabling characterization of upper-body orientation and asymmetry.

All trajectories were temporally filtered as described in Section 2.5. Analysis was restricted to short temporal segments characterized by minimal voluntary movement, in order to isolate postural control from preparatory actions.

To identify these intervals, a sliding-window procedure was applied to the vertical trajectory of the neck KeyPoint. The window minimizing the combined root-mean-square of vertical velocity and acceleration was selected, corresponding to the phase of lowest corrective effort immediately preceding propulsion.

Within this window, kinematic descriptors were extracted to quantify short-term postural steadiness, upper-body compensatory strategies, and proxy indicators of buoyancy maintenance under aquatic constraints. The full list of features is reported in Supplementary Tables S5–S7.

#### 2.9.2. Static Floating Index (SFI)

To obtain a compact descriptor of performance during the upright floating task, the extracted kinematic features were aggregated into a Static Floating Index (SFI), summarizing the athlete’s ability to maintain a stable upright posture with minimal corrective effort. Two complementary formulations were defined:

• SFI_core, based on a reduced subset of descriptors capturing the main dimensions of postural control, including vertical steadiness, horizontal sway, alignment stability, and corrective smoothness
• SFI_ext, based on the full feature set, including additional descriptors of upper-body compensatory strategies and proxy indicators of buoyancy maintenance

The extended formulation was not intended as a primary classification-oriented summary, but rather as a complementary descriptor to support biomechanical interpretation of individual stabilization strategies.

Feature values were first aligned in physiological directionality so that higher values consistently reflected poorer postural control. To enable comparison across subjects, individual descriptors were then scaled using fixed bounded reference intervals defined at the feature level, rather than subject-specific normalization across repeated trials.

The SFI was computed by aggregating available scaled descriptors only, automatically excluding missing values without imputation. This approach preserves interpretability while avoiding bias due to incomplete tracking.

In addition to the global composite scores, domain-specific summaries were derived to support interpretation of the main contributors to performance, including core postural stability, head–trunk coordination, upper-body compensatory behavior, and buoyancy-maintenance effort.

Overall, the SFI provides an objective and interpretable summary of short-term postural control during upright floating, supporting both between-subject comparison and the identification of compensatory stabilization strategies.

### 2.10. Explosive Vertical Propulsion from Upright Floating

Explosive vertical propulsion is a key functional requirement in water polo, as it enables athletes to elevate the upper body above the water surface for passing, shooting, and defensive actions. This ability depends on the coordinated contribution of lower-limb propulsion, trunk stabilization, and, when permitted, upper-limb involvement.

To distinguish different mechanical contributions to this task, two experimental conditions were analyzed: a combined propulsion condition (legs + arms), representing whole-body force generation under ecologically relevant game-like conditions, and a legs-only propulsion condition, designed to isolate lower-limb contribution by minimizing the role of the upper limbs.

From a functional perspective, this task probes not only the capacity to generate rapid upward displacement, but also the ability to control body alignment and recover postural stability after thrust. The comparison between the two conditions further supports interpretation of segmental contribution and compensatory strategy, which is particularly relevant in athletes with upper-limb impairment.

In the present framework, explosive vertical propulsion was therefore analyzed as a task-specific probe of propulsion capacity and stabilization under different mechanical constraints

#### 2.10.1. Feature Extraction

Explosive vertical propulsion was analyzed using three-dimensional trajectories of upper-body KeyPoints that remained consistently visible above the water surface. The analysis focused on the neck (BO01) as the primary reference for vertical displacement, the nose (BO00) to support head-level tracking, and the left and right shoulders (BO05, BO02) to characterize trunk contribution and symmetry during ascent. In the combined propulsion condition, elbows and wrists were additionally considered when sufficiently visible in order to quantify upper-limb contribution to propulsion.

All trajectories were temporally filtered as described in Section 2.5. Feature extraction was performed within event-centered propulsion windows aligned to the onset of upward motion, identified from the vertical velocity profile of the neck KeyPoint. For each trial, propulsion onset was defined as the first frame in which vertical velocity exceeded a task-specific threshold after a stable pre-thrust interval, and the propulsion phase extended until peak vertical displacement was reached.

Kinematic descriptors were extracted to quantify propulsion capacity, temporal efficiency, trunk stabilization, and segmental coordination. In the combined propulsion condition, additional variables were used to characterize upper-limb contribution relative to trunk motion. In the legs-only condition, feature extraction was restricted to head and trunk kinematics so that the resulting descriptors primarily reflected lower-limb propulsion and post-thrust stabilization.

The full list of extracted features is reported in Supplementary Tables S8–S10.

#### 2.10.2. Vertical Propulsion Index (VPI)

To obtain a compact descriptor of explosive vertical propulsion performance, the extracted features were aggregated into a Vertical Propulsion Index (VPI), summarizing the athlete’s ability to generate rapid and effective upward displacement while maintaining postural control.

Two condition-specific formulations were defined:

• VPI_LA, corresponding to the combined propulsion condition (legs + arms), intended to capture whole-body propulsion under ecologically relevant conditions
• VPI_L, corresponding to the legs-only condition, intended to isolate lower-limb contribution under constrained mechanical conditions

Both formulations were based on descriptors of propulsion capacity, temporal efficiency, and post-thrust stabilization. Feature values were aligned in physiological directionality so that higher index values consistently reflected poorer functional performance.

Rather than using subject-level normalization across repeated trials, descriptors were transformed using fixed impairment-oriented scaling rules defined at the feature level. This strategy preserves direct comparability across subjects while maintaining the interpretation of absolute performance differences under both propulsion conditions.

The VPI was computed by aggregating available scaled descriptors only, automatically excluding missing values without imputation.

The distinction between VPI_LA and VPI_L supports interpretation of segmental contribution and compensatory strategy. In particular, deterioration in the legs-only condition may indicate reliance on upper-limb assistance, whereas comparable performance across conditions may reflect preserved lower-limb propulsion and trunk-mediated control.

#### 2.10.3. Cross-Condition Functional Interpretation

The simultaneous availability of propulsion performance in the combined (legs + arms) and constrained (legs-only) conditions enables task-specific interpretation of functional ability under different mechanical demands.

Although both conditions were described within the same general feature framework, the underlying segmental contributions differed substantially. The combined condition reflects whole-body propulsion under ecologically relevant constraints, whereas the legs-only condition more specifically probes lower-limb propulsion and trunk-mediated stabilization.

Comparison between conditions therefore provides indirect information on segmental contribution and compensatory strategy. In particular, deterioration in the legs-only condition may indicate reliance on upper-limb assistance, whereas comparable performance across conditions may reflect preserved lower-limb propulsion and effective trunk control.

Accordingly, the two VPI formulations are not intended as isolated performance scores, but as complementary descriptors supporting interpretation of how explosive vertical propulsion is achieved under different mechanical constraints. This distinction is especially relevant in Paralympic sport, where similar task outcomes may arise from different underlying motor strategies.

### 2.11. Ball Passing

Passing is a fundamental upper-limb action in water polo, requiring the coordinated generation and transmission of force from the trunk to the distal arm segments, followed by effective ball release. Successful execution depends not only on residual arm function, but also on the ability to organize posture, prepare the movement, and transfer propulsion through a stable and well-timed proximal-distal sequence.

From a functional perspective, passing provides a task-specific probe of upper-limb residual capacity, release organization, and compensatory trunk involvement. In athletes with impairment, it is particularly informative because it reveals how residual arm function, postural adjustments, and whole-body contribution are combined to achieve effective ball propulsion.

In the present protocol, passing was evaluated separately for the right and left arms, enabling side-specific characterization of functional capacity and inter-limb asymmetry under controlled task conditions.

#### 2.11.1. Feature Extraction

Passing was analyzed using three-dimensional trajectories of upper-body and upper-limb KeyPoints directly involved in force generation, transmission, and release. The selected landmarks included the neck (BO01) and shoulders (BO02, BO05), defining the trunk reference frame, together with the elbow and wrist of the active arm (BO03–BO04 or BO06–BO07), capturing upper-limb propulsion and coordination.

For ball-related tasks, human KeyPoint analysis was complemented by frame-wise ball detection. The ball was localized using a YOLO-based detector, and the release event was identified automatically by combining ball localization with the active-hand trajectory. Release timing was defined as the frame at which the ball ceased to be in contact with the active hand and began an independent trajectory, as supported by the spatial relationship between the ball and distal upper-limb KeyPoints across adjacent frames.

All trajectories were temporally filtered as described in Section 2.5. In contrast with the previous onset-threshold approach, feature extraction for passing was performed within a release-centered framework. Once ball release had been identified, descriptors were computed in fixed temporal windows referenced to this event. When applicable, a pre-release window, a release-centered window, and a post-release recovery window were considered. This strategy reduced dependence on variable preparation duration and follow-through length, while improving biomechanical interpretability and comparability across trials and subjects.

Kinematic descriptors were extracted to quantify:

• postural control and release geometry during passing,
• upper-limb propulsion and temporal organization,
• proximal-distal coordination, and
• compensatory trunk involvement during force transmission.

Additional inter-limb comparison descriptors were derived to support interpretation of asymmetries in side-specific passing performance.

The full list of extracted features is reported in Supplementary Tables S11–S14.

#### 2.11.2. Passing Arm Residual Index (PARI)

To obtain a compact descriptor of upper-limb functional performance during passing, the extracted features were aggregated into a Passing Arm Residual Index (PARI), computed separately for the right (PARI_R) and left (PARI_L) arms.

The PARI summarizes the ability of the active limb to generate and transmit propulsion during passing while preserving sufficient coordination, release organization, and postural control. In addition to the composite descriptors, domain-specific components were considered to separately characterize (i) output/release effectiveness, (ii) upper-limb coordination and timing, (iii) postural control, and (iv) compensatory strategy during the task.

Feature values were first aligned in physiological directionality so that higher index values consistently reflected less favorable functional performance. Rather than relying on within-subject normalization across repeated trials, descriptors were transformed using fixed impairment-oriented scaling rules, thereby preserving direct comparability across subjects and maintaining the interpretation of absolute performance differences.

The index was computed using only available valid descriptors, without imputation. When tracking quality was locally insufficient, only the most tracking-sensitive variables were excluded from scoring, while the remaining valid descriptors for that trial were retained. This feature-level quality-control approach preserved interpretability while limiting bias due to partial missingness.

The arm-specific formulation of the PARI supports the identification of functional asymmetries and compensatory strategies, providing a structured summary of residual upper-limb capacity during a task directly relevant to gameplay.

#### 2.11.3. Inter-Limb Functional Asymmetry

To further characterize arm-specific differences in passing performance, additional descriptors were computed by comparing right and left arm conditions within each subject.

These descriptors quantified asymmetries in propulsion capacity, release organization, postural control, and coordination, and were not included in the computation of the PARI. Instead, they were used as complementary outputs to support interpretation of inter-limb differences and compensatory strategies.

The full list of comparison descriptors is reported in Supplementary Table S14.

### 2.12. Non-Contested Shooting

Non-contested shooting represents a controlled task condition in which the athlete can organize posture, arm motion, and ball release without external physical perturbation. As such, it provides a reference framework for evaluating shooting-specific upper-limb function under stable task conditions, while preserving the ecological structure of a sport-relevant action.

From a functional perspective, non-contested shooting probes the ability to generate effective ballistic output and release the ball from an adequate shooting position, while maintaining sufficient postural control to support force transmission throughout the action. The task is therefore informative not only for upper-limb propulsion, but also for release geometry, preparatory movement organization, and whole-body involvement during the shot.

In the present protocol, shooting performance was evaluated separately for the right and left arms, enabling side-specific characterization of residual functional capacity under stable conditions and allowing inter-limb comparisons within each subject.

#### 2.12.1. Feature Extraction

Non-contested shooting was analyzed using three-dimensional trajectories of upper-body and upper-limb KeyPoints involved in force generation, transmission, and release. The selected landmarks included the neck (BO01) and shoulders (BO02, BO05), defining the trunk reference frame, together with the elbow and wrist of the active arm (BO03–BO04 or BO06–BO07), capturing upper-limb propulsion and coordination.

All trajectories were temporally filtered as described in Section 2.5. In contrast with the previous event-threshold approach, feature extraction was performed in a release-centered framework. Ball release timing was first identified using the same automatic ball-processing procedure described for the passing task, based on YOLO ball detection and the frame-wise relationship between the ball and the active hand, and all descriptors were then computed in fixed temporal windows referenced to this event. Specifically, three regions were considered when applicable: a pre-release window, a release-centered window, and a post-release recovery window. This strategy reduced dependence on variable preparation duration, follow-through duration, and partial tracking loss, while improving biomechanical interpretability across trials and subjects.

The extracted descriptors quantified three complementary aspects of the shooting action:

• postural control and release geometry, including trunk stability around release, release height, and postural cost of shooting.
• upper-limb explosive output and coordination, including peak wrist speed, peak wrist acceleration, release-timing organization, and proximal-distal coordination; and
• preparatory and compensatory strategy, including trunk involvement, rotational contribution, and pre-release loading organization.

To better capture different shooting strategies, preparatory loading was not treated as a single pattern. Instead, two complementary loading families were considered: horizontal loading, reflecting broader cocking actions with more evident pre-release backward preparation, and compact loading, reflecting shorter and more localized preparatory phases that still showed a structured pre-release reorganization. This distinction was introduced to accommodate subject-specific motor solutions, including powerful shots executed from relatively low release positions or with compact pre-release organization.

Kinematic descriptors were therefore extracted to quantify ballistic output, release height, preparatory structure, proximal-distal sequencing, and postural cost under shooting-specific mechanical demand. The full list of extracted features is reported in Supplementary Tables S15–S17.

#### 2.12.2. Shooting Arm Residual Index (SARI)

To obtain a compact descriptor of shooting-specific upper-limb function, the extracted features were aggregated into a Shooting Arm Residual Index (SARI), computed separately for the right (SARI_R) and left (SARI_L) arms. The SARI summarizes the quality of task execution during non-contested shooting by integrating release-centered descriptors of ballistic output, release geometry, upper-limb coordination, and postural control. In addition to composite indices, domain-specific descriptors were also derived to separately characterize propulsion/timing, upper-limb coordination, and postural control.

Feature values were first aligned in physiological directionality so that higher scores consistently reflected less favorable functional performance. Unlike the previous within-subject normalization scheme, descriptors were transformed using fixed, impairment-oriented scaling rules, allowing direct comparability across subjects while preserving the meaning of absolute performance differences.

In the final composite formulation, the SARI was implemented as an output-weighted index, giving greater emphasis to ballistic execution and release configuration while retaining complementary descriptors of coordination and postural control. To avoid overemphasizing superficial differences in preparatory style, loading-related descriptors were incorporated as a single loading-evidence component, combining horizontal and compact loading information into one release-preparation construct. In addition, a contextual lag component was retained to describe the temporal relationship between distal output development and ball release within the release-centered framework.

The index was computed using only available valid descriptors, without imputation. When tracking quality was locally insufficient, only the most tracking-sensitive variables were excluded from scoring, while the remaining valid descriptors for that trial were retained. This feature-level quality-control approach preserved interpretability while reducing bias due to partial missingness.

Compared with the passing task, the SARI for non-contested shooting places greater emphasis on ballistic execution, release configuration, and stability under load, thereby providing a complementary summary of residual upper-limb function in a task more directly linked to scoring performance.

### 2.13. Contested Shooting Under Physical Opposition

In contrast to non-contested shooting, this task was designed to evaluate the athlete’s ability to tolerate direct physical opposition and reorganize motor behavior in order to complete, or attempt to complete, a ball release under perturbation. The presence of an opponent introduces ecologically relevant constraints that affect buoyancy control, upright stability, and upper-limb force transmission.

From a functional perspective, contested shooting probes not only residual shooting capacity, but also robustness to perturbation and efficiency of post-contact recovery. This distinction is particularly relevant in Paralympic sport, where similar performance outcomes may be achieved through different underlying motor strategies and where functional limitations may emerge primarily under externally imposed constraints [1].

#### 2.13.1. Feature Extraction

Contested shooting was analyzed using a phase-based representation aligned with the intrinsic discontinuities of the task. Unlike non-contested shooting, physical opposition frequently resulted in partial or complete loss of KeyPoint visibility, requiring segmentation criteria based on both kinematic structure and upper-body visibility.

Three functional phases were defined: pre-contact, corresponding to stable upright control before physical interaction; contact/submersion, corresponding to the interval of perturbation and visibility loss; and post-contact, corresponding to re-emergence and motor reorganization leading to a shooting attempt.

Phase boundaries were identified from the temporal continuity of head and trunk KeyPoints, primarily the nose and neck. The onset of the contact phase was defined as the last valid frame preceding sustained upper-body visibility loss, whereas re-emergence was defined as the first frame with restored upper-body visibility.

In addition to phase-based segmentation, ball release was identified automatically whenever a reliable post-contact release event could be detected. Release timing was determined using the same YOLO-based ball detection and ball–hand separation logic adopted for the other ball-related tasks. This information was not used to redefine the primary task phases, but to refine the characterization of post-contact execution, release geometry, and action outcome, and to support comparisons with the non-contested shooting condition.

Feature extraction was restricted to temporally contiguous segments with reliable tracking. No interpolation was applied during submersion or partial occlusion, in order to preserve the functional meaning of discontinuous observability.

Descriptors were extracted to quantify perturbation tolerance, recovery dynamics, and residual shooting performance during the post-contact phase. These included event- and outcome-based variables, early reorganization descriptors, and post-contact shooting measures comparable with the non-contested condition when a valid release was available.

The full list of extracted features is reported in Supplementary Tables S18–S20.

#### 2.13.2. Contested Shooting Resistance Index (CSRI)

To obtain a compact descriptor of performance under physical perturbation, the extracted features were aggregated into a Contested Shooting Resistance Index (CSRI), computed separately for the right (CSRI_R) and left (CSRI_L) arms. Unlike indices derived from continuous kinematic tasks, the CSRI was conceived as a resistance-and-reorganization descriptor explicitly accounting for the intrinsic discontinuity of contested actions. The index summarizes the athlete’s ability to tolerate perturbation, recover an upright functional posture, and reorganize movement toward a shooting attempt.

Two complementary formulations were defined:

• CSRI_core, based on robust event- and outcome-based variables, including submersion time, re-emergence success, post-contact preparation time, and task-completion descriptors.
• CSRI_ext, incorporating additional post-contact kinematic descriptors related to recovery quality and residual shooting performance, together with release-centered post-contact descriptors when a valid release was available.

Feature values were first aligned in physiological directionality so that higher index values consistently reflected poorer functional performance. Rather than using subject-level normalization across repeated trials, descriptors were transformed using fixed impairment-oriented scaling rules, thereby preserving direct comparability across subjects while maintaining the interpretation of absolute performance differences under contested conditions.

The index was computed by aggregating only the available valid scaled descriptors, automatically excluding missing values without imputation. This approach is particularly important in contested conditions, where temporary loss of observability is intrinsic to task execution and carries functional meaning.

Overall, the CSRI provides a structured and interpretable summary of perturbation tolerance and post-contact motor reorganization, complementing continuous-task indices with a task-specific descriptor of residual function under ecologically valid opposition.

#### 2.13.3. Derived Integrated Descriptors (Tier 4)

In addition to the primary event-based and kinematic descriptors, a set of derived integrated metrics was defined to capture higher-level functional relationships across phases of the contested shooting task.

These descriptors combined information from different phases of the analysis pipeline and were classified as Tier 4 integrated descriptors. They were not included in the computation of the CSRI but were used as complementary outputs to support interpretation of performance under perturbation.

The following descriptors were defined:

• Recovery Efficiency Index (REI): ratio between peak wrist speed and the sum of submersion time and post-contact preparation time, reflecting the efficiency with which post-perturbation recovery is translated into effective distal propulsion.
• Compensation Burden Index (CBI): product of trunk dominance at release and the sum of submersion time and post-contact preparation time, reflecting the overall compensatory burden associated with delayed recovery, increased trunk involvement, and task completion under perturbation.

Higher REI values indicate a faster and more efficient transition from re-emergence to shooting execution, whereas higher CBI values indicate greater reliance on compensatory whole-body strategies associated with delayed recovery and increased trunk involvement.

The full list and definition of derived integrated descriptors are reported in Supplementary Table S21. Although expressed in derived units, these quantities should be interpreted as composite functional descriptors rather than direct physical measures, because they combine variables with different biomechanical meanings.

#### 2.13.4. Comparison Between Non-Contested and Contested Shooting Performance

In addition to task-specific descriptors, performance during the post-contact phase of contested shooting was compared with the corresponding non-contested shooting condition.

This comparison was performed only when a reliable and temporally contiguous post-contact shooting window could be identified following re-emergence. For release-based variables, comparison was restricted to trials in which a valid post-contact ball release was available. Trials without release or with insufficient tracking continuity were excluded from release-centered comparison.

The purpose of this comparison was not to assume a uniform degradation of shooting performance under perturbation, but to contextualize how baseline shooting capacity was preserved, modified, or reorganized after physical opposition. Whereas non-contested shooting provided a reference measure of upper-limb propulsion, release organization, and coordination under stable conditions, contested shooting reflected how this capacity was re-expressed following submersion and recovery.

Only descriptors defined consistently in both conditions and computed using comparable segmentation and processing rules were considered. When appropriate, contested post-contact descriptors were aligned to the release event in the same way as in the non-contested condition. These included measures of propulsion capacity, temporal organization, release geometry, intersegmental coordination, and trunk contribution during shooting.

Absolute differences and relative ratios were derived on a within-subject and within-arm basis to quantify performance degradation, preservation, or compensatory reorganization under perturbation. Accordingly, this comparison should be interpreted as a descriptor of conditional post-contact execution relative to non-contested shooting, rather than as a pure degradation score for the contested task as a whole.

The full list of comparison descriptors is reported in Supplementary Table S22.

## 3. Results

### 3.1. Subject-Specific Upright Floating Profiles

**Figure 3:**
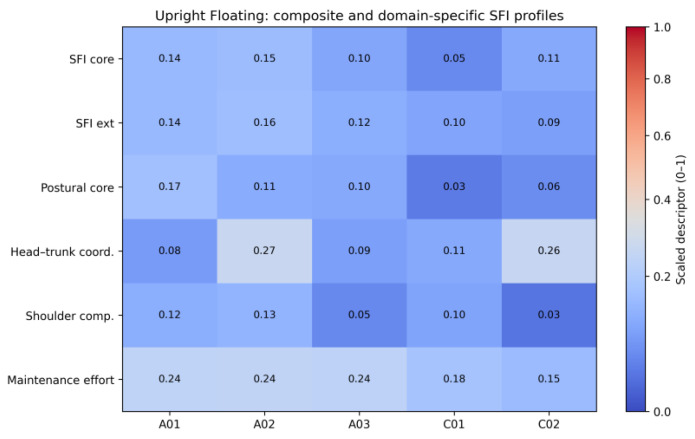
Heatmap of composite and domain-specific SFI descriptors during Upright Floating.

**Figure 4:**
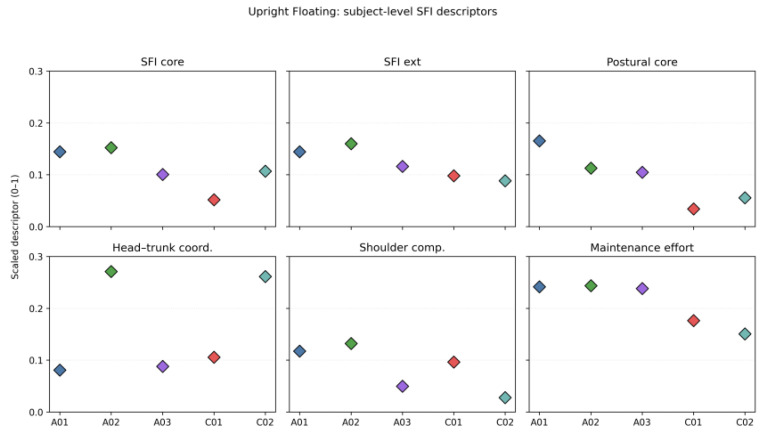
Subject-level composite and domain-specific SFI descriptors during Upright Floating. Graphical distribution of subject-level Static Floating Index (SFI) composite scores and domain-specific components during the upright floating task. Each diamond represents one participant, with marker colors used only to facilitate visual distinction among subjects. SFI_core and SFI_ext denote the core and extended Static Floating Index formulations, respectively. Higher scaled values indicate greater task-specific functional burden.

Results from the Upright Floating task showed generally low scaled descriptor values across the sample, with SFI_core values ranging from 0.05 to 0.15. Subject-specific differences nevertheless emerged when the global composite descriptors were considered together with the domain-specific components, as summarized in Table 3 and visualized in Figure 3 and Figure 4.

Within the subgroup of athletes with eligible impairments, A02 showed the highest global composite values, with SFI core = 0.15 and SFI ext = 0.16, together with a particularly elevated value in the head–trunk coordination domain (0.27). By contrast, A03 showed the lowest global composite values within the subgroup of athletes with eligible impairments (SFI core = 0.10; SFI ext = 0.12) and the lowest shoulder-compensation value (0.05), whereas its maintenance-effort value was identical to those observed for A01 and A02 (0.24). A01 showed SFI_core and SFI_ext values of 0.14, together with the highest postural-core value within the subgroup of athletes with eligible impairments (0.17) and the lowest head–trunk coordination value (0.08).

The two able-bodied reference participants showed relatively low global composite values, although with different distributions across the domain-specific components. C01 showed the lowest SFI core value (0.05) and the lowest postural-core value (0.03) across the sample. C02 showed the lowest SFI ext value (0.09), together with the lowest shoulder-compensation (0.03) and maintenance-effort (0.15) values, but showed a comparatively higher head–trunk coordination value (0.26), similar to that observed in A02. As illustrated in Figure 3, subjects with relatively low global composite values nevertheless showed different distributions across the domain-specific components.

Taken together, Table 3 and Figure 3 and Figure 4 highlight distinct stabilization strategies across subjects and support a descriptive comparison of task-specific functional profiles rather than a rank-based interpretation.

### 3.2. Subject-Specific Explosive Vertical Propulsion Profiles (Legs + Arms)

**Figure 5:**
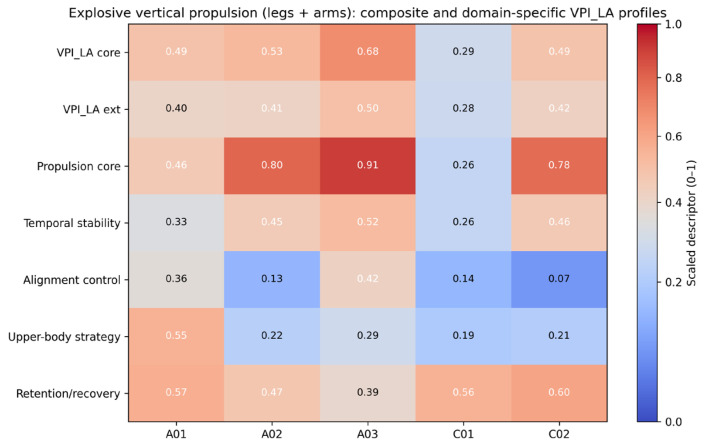
Heatmap of composite and domain-specific VPI_LA descriptors during Explosive Vertical Propulsion (legs + arms).

**Figure 6:**
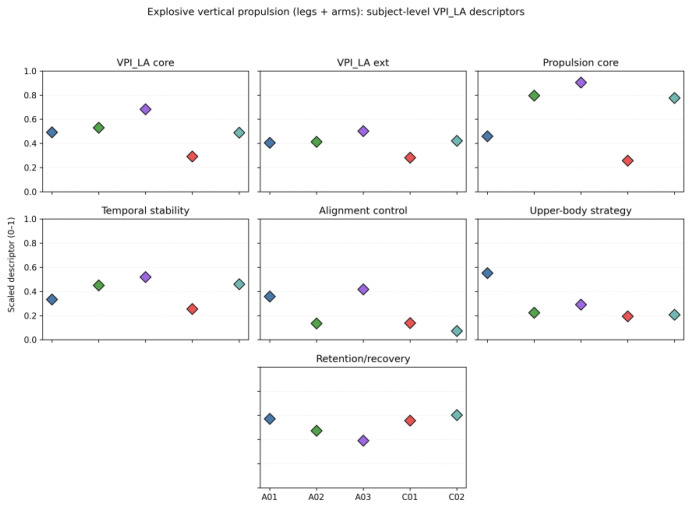
Subject-level composite and domain-specific VPI_LA descriptors during Explosive Vertical Propulsion (legs + arms).Graphical distribution of subject-level Vertical Propulsion Index descriptors for the combined legs + arms condition (VPI_LA). Each diamond represents one participant, with marker colors used only to facilitate visual distinction among subjects. VPI_LA_core and VPI_LA_ext denote the core and extended formulations of the Vertical Propulsion Index for the combined propulsion condition, respectively. Higher scaled values indicate greater task-specific functional burden.

Results from the Explosive Vertical Propulsion (legs + arms) task showed a wider spread of subject-level composite values than Upright Floating, with VPI_LA_core ranging from 0.29 to 0.68 and VPI_LA_ext from 0.28 to 0.50 (Table 4; Figure 5 and Figure 6).

Within the subgroup of athletes with eligible impairments, A03 showed the highest VPI_LA_core (0.68) and VPI_LA_ext (0.50) values. A03 also showed the highest propulsion-core (0.91), temporal-stability (0.52), and alignment-control (0.42) values across the sample. A02 showed a propulsion-core value of 0.80, together with lower alignment-control (0.13) and upper-body-strategy (0.22) values. A01 showed VPI_LA_core and VPI_LA_ext values of 0.49 and 0.40, respectively, together with the highest upper-body-strategy value across the sample (0.55) and a retention/recovery value of 0.57.

The two able-bodied reference participants showed non-identical global composite and domain-specific profiles. C01 showed the lowest VPI_LA_core (0.29) and VPI_LA_ext (0.28) values, together with the lowest propulsion-core (0.26) and temporal-stability (0.26) values across the sample.

C02 showed VPI_LA_core and VPI_LA_ext values of 0.49 and 0.42, respectively, together with propulsion-core and temporal-stability values of 0.78 and 0.46. It also showed the lowest alignment-control value (0.07) and the highest retention/recovery value (0.60) across the sample.

Taken together, Table 4 and Figure 5 and Figure 6 show distinct subject-specific distributions across the global composite and domain-specific VPI_LA descriptors.

### 3.3. Subject-Specific Explosive Vertical Propulsion Profiles (Legs)

**Figure 7:**
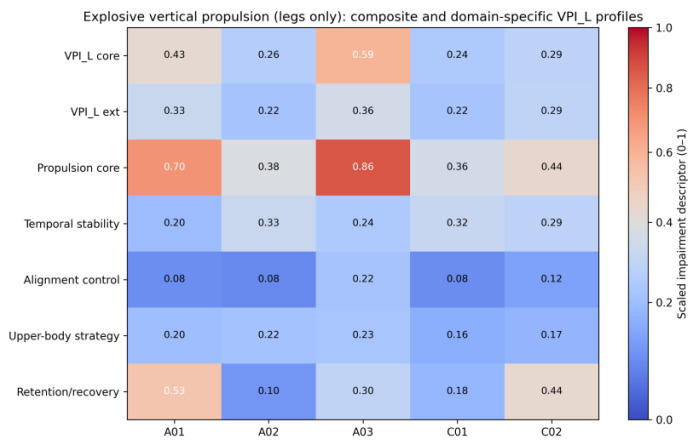
Heatmap of composite and domain-specific VPI_L profiles during vertical propulsion with legs only. Higher scaled values indicate greater task-specific functional burden.

**Figure 8:**
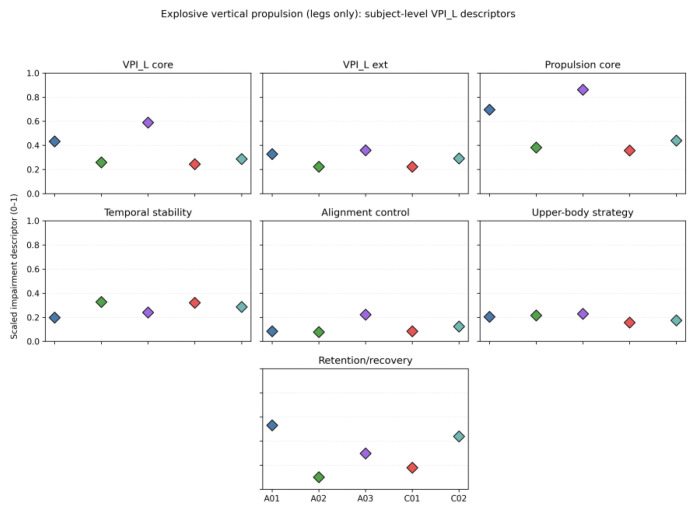
Subject-level distribution of composite and domain-specific VPI_L descriptors during vertical propulsion with legs only. Higher scaled values indicate greater task-specific functional burden.

Subject-level VPI_L profiles for the explosive vertical propulsion task performed with legs only are reported in Table 5, while the corresponding heatmap and domain-specific graphical distributions are shown in Figure 7 and Figure 8, respectively.

Across the sample, VPI_L_core values ranged from 0.24 to 0.59 and VPI_L_ext values from 0.22 to 0.36. A03 showed the highest composite values (VPI_L_core = 0.59; VPI_L_ext = 0.36), whereas C01 showed the lowest values (VPI_L_core = 0.24; VPI_L_ext = 0.22). A01 showed VPI_L_core and VPI_L_ext values of 0.43 and 0.33, respectively. A02 showed corresponding values of 0.26 and 0.22, which were close to those observed in C01 (0.24 and 0.22) and lower than those observed in C02 (0.29 and 0.29). C02 showed VPI_L_core and VPI_L_ext values of 0.29 and 0.29, respectively, both higher than the corresponding values observed in C01 (0.24 and 0.22).

Across subjects, propulsion-core values ranged from 0.36 to 0.86. They represented the highest domain-specific values for A01, A02, A03, and C01 and were tied with retention/recovery in C02. The values were 0.86 for A03, 0.70 for A01, 0.44 for C02, 0.38 for A02, and 0.36 for C01 (Table 5; Figure 7 and Figure 8). Alignment-control values ranged from 0.08 to 0.22, with values of 0.08 for A01, A02, and C01, 0.12 for C02, and 0.22 for A03. Temporal-stability values were 0.33 for A02, 0.32 for C01, 0.29 for C02, 0.24 for A03, and 0.20 for A01. Upper-body-strategy values ranged from 0.16 to 0.23 across the sample. The highest retention/recovery values were observed in A01 (0.53) and C02 (0.44), followed by A03 (0.30), C01 (0.18), and A02 (0.10).

Among the domain-specific descriptors, propulsion-core values showed the widest range across subjects (0.36–0.86), whereas alignment-control and upper-body-strategy values ranged from 0.08 to 0.22 and from 0.16 to 0.23, respectively. Among the athletes with eligible impairments, VPI_L_core values were highest for A03 (0.59), followed by A01 (0.43) and A02 (0.26); VPI_L_ext values followed the same ordering (0.36, 0.33, and 0.22, respectively) (Table 5; Figure 7 and Figure 8).

### 3.4. Subject-Specific Ball Passing Profiles

**Figure 9:**
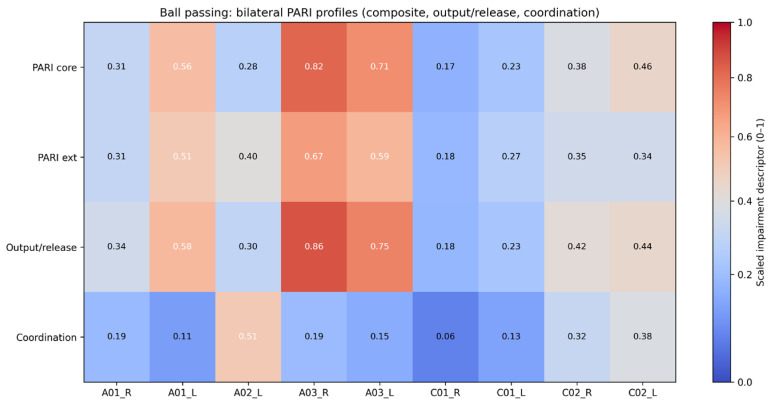
Bilateral heatmap of subject-level PARI descriptors for the ball passing task. Each column represents one subject-side combination, and rows report the composite indices (PARI core and PARI ext) together with the output/release and coordination domains. Higher values indicate greater task-specific functional burden.

**Figure 10:**
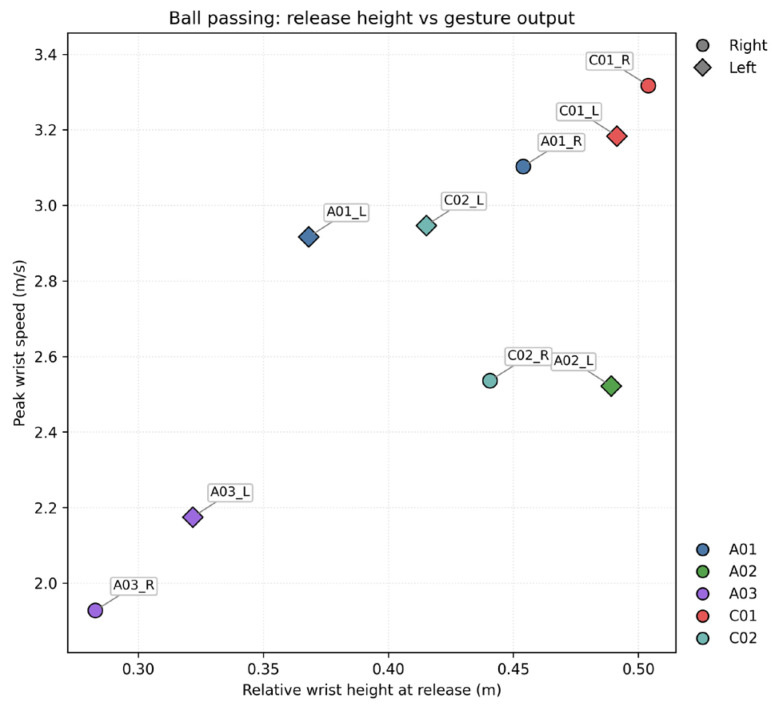
Subject-side distribution of relative wrist height at release versus peak wrist speed during the ball passing task. Colors identify subjects and marker shapes distinguish sides. Higher values on the two axes correspond to a higher release position and greater peak wrist speed, respectively.

Subject-specific bilateral PARI profiles for the ball passing task are summarized in Table 6. The corresponding bilateral distribution of composite and domain-specific descriptors is shown in Figure 9, while Figure 10 reports the relationship between relative wrist height at release and peak wrist speed for each available subject–side combination.

Across the available subject–side combinations, PARI_core values ranged from 0.167 to 0.819, whereas PARI_ext values ranged from 0.181 to 0.668. A01 showed higher values for the left arm than for the right arm, with PARI_core values of 0.557 and 0.308 and PARI_ext values of 0.509 and 0.308, respectively. The corresponding left-minus-right asymmetries were 0.249 for PARI_core and 0.200 for PARI_ext.

A02 performed the task only with the left arm and showed PARI_L_core and PARI_L_ext values of 0.277 and 0.398, respectively. A03 showed the highest bilateral composite values across the sample. On the right side, PARI_R_core and PARI_R_ext were 0.819 and 0.668, respectively, whereas the corresponding left-side values were 0.713 and 0.588. This resulted in left-minus-right asymmetries of −0.106 for PARI_core and −0.079 for PARI_ext.

Among the able-bodied reference participants, C01 showed PARI_R_core and PARI_R_ext values of 0.167 and 0.181 and PARI_L_core and PARI_L_ext values of 0.229 and 0.270. C02 showed corresponding right-side values of 0.378 and 0.347 and left-side values of 0.456 and 0.344. The core asymmetry was 0.063 for C01 and 0.078 for C02, whereas the extended asymmetry was 0.089 for C01 and −0.002 for C02.

Figure 9 shows the distribution of the composite indices together with the output/release and coordination descriptors. A03 showed the highest bilateral composite values and comparatively high output/release values. A01 showed higher composite and output/release values on the left than on the right. For A02, only the left-side profile was available. C01 showed the lowest bilateral composite values, whereas C02 showed values between those observed for C01 and A03. Postural-control and compensatory-strategy descriptors were retained in the analysis but were not included in the main heatmap.

Figure 10 provides the release-centered distribution of relative wrist height and peak wrist speed. The C01 subject–side points were located toward higher values on both axes, whereas the A03 points were located toward lower release-height and peak-wrist-speed values. The right- and left-side points of A01 occupied different positions, with the left side showing lower values than the right. The A02 left-side and C02 bilateral points were positioned between these distributions.

### 3.5. Subject-Specific Ball Shooting Profiles

**Figure 11:**
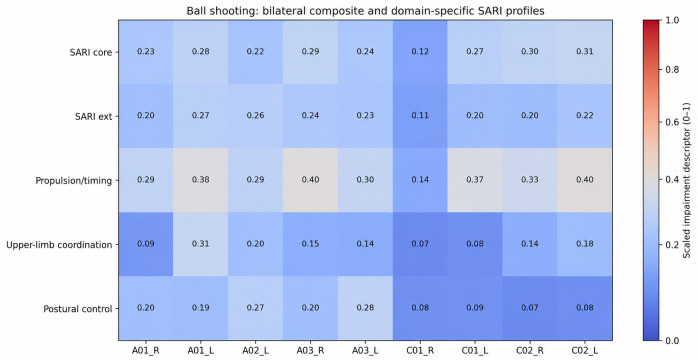
Heatmap of bilateral composite and domain-specific SARI profiles during the ball shooting task. Each row represents one subject-side profile. Higher scaled values indicate greater task-specific functional burden.

**Figure 12:**
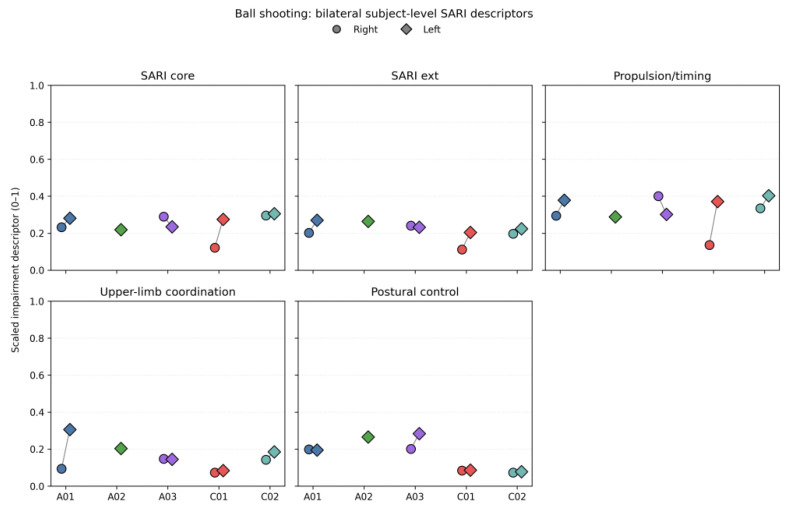
Bilateral subject-level distribution of composite and domain-specific SARI descriptors during the ball shooting task. Right and left arm values are shown separately to highlight within-subject asymmetry. Higher scaled values indicate greater task-specific functional burden.

**Figure 13:**
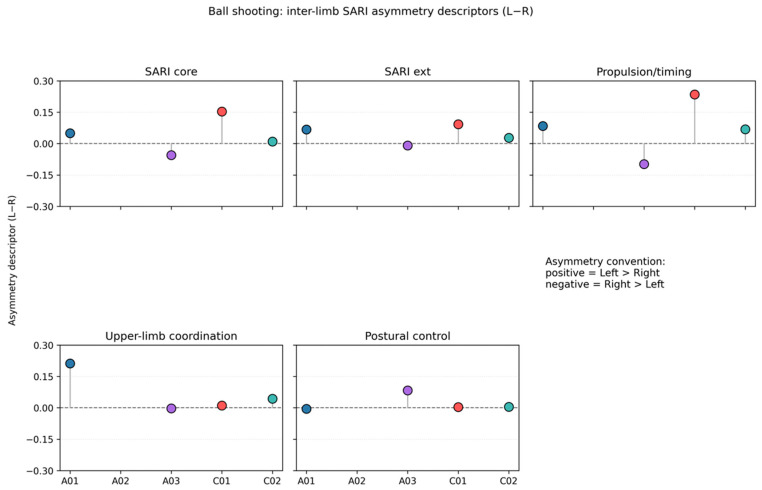
Inter-limb asymmetry descriptors (L−R) for bilateral SARI composite and domain-specific indices during the ball shooting task. Positive values indicate greater task-specific functional burden on the left side, whereas negative values indicate greater impairment on the right side.

**Figure 14:**
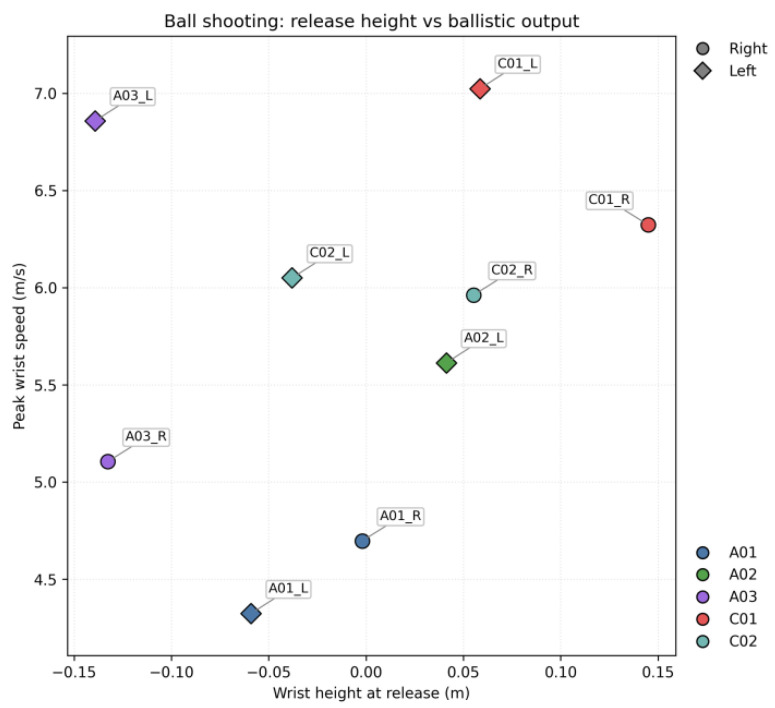
Subject-level relationship between release height and ballistic output during the ball shooting task. Each marker represents one subject-side profile, plotted according to wrist height at release and peak wrist speed. This representation highlights differences in shooting strategy and performance, distinguishing subjects who release from a higher position from those who achieve greater ballistic output despite lower release height.

**Figure 15:**
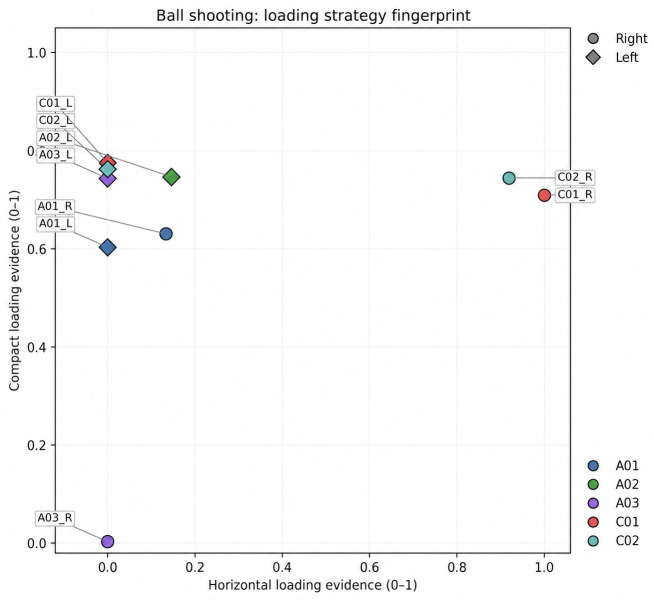
Subject-level loading-strategy fingerprint during the ball shooting task. Each marker represents one subject-side profile, plotted according to horizontal loading evidence and compact loading evidence. This representation distinguishes different preparatory shooting strategies, separating profiles characterized by broad horizontal cocking actions from those relying on shorter, more compact pre-release loading patterns.

Subject-specific bilateral SARI profiles for the ball shooting task are summarized in Table 7. The corresponding heatmap, paired right-left distributions, and inter-limb asymmetry descriptors are shown in Figure 11, Figure 12 and Figure 13, respectively. Additional bivariate representations of release height and ballistic output and of pre-release loading are reported in Figure 14 and Figure 15.

Across the available subject-side combinations, SARI_core values ranged from 0.122 to 0.306, whereas SARI_ext values ranged from 0.112 to 0.270. A01 showed higher composite values for the left arm than for the right arm, with SARI_core values of 0.282 and 0.232 and SARI_ext values of 0.270 and 0.202, respectively. The corresponding left-minus-right differences were 0.050 for SARI_core and 0.068 for SARI_ext.

A02 performed the task only with the left arm and showed SARI_L_core and SARI_L_ext values of 0.219 and 0.264, respectively. A03 showed lower composite values on the left than on the right, with SARI_L_core and SARI_L_ext values of 0.235 and 0.232 and corresponding right-side values of 0.290 and 0.241. This resulted in left-minus-right differences of −0.054 for SARI_core and −0.009 for SARI_ext.

Among the able-bodied reference participants, C01 showed SARI_R_core and SARI_R_ext values of 0.122 and 0.112, respectively, and left-side values of 0.275 and 0.204. C02 showed corresponding right-side values of 0.296 and 0.197 and left-side values of 0.306 and 0.224.

The domain-specific distributions in Figure 11 and Figure 12 showed non-identical patterns across subjects and sides. In A01, the left-side values exceeded the corresponding right-side values particularly in the propulsion/timing and upper-limb coordination components. In A03, the left-side propulsion/timing value was lower than the corresponding right-side value, whereas the postural-cost component remained comparatively higher. For A02, the available left-side profile showed a lower core than extended composite value. C01 showed a larger between-side difference than C02, particularly in the core composite and propulsion-related components.

The asymmetry descriptors reported in Figure 13 quantify these bilateral differences. A01 showed positive left-minus-right asymmetries for both SARI_core (0.050) and SARI_ext (0.068), whereas A03 showed negative asymmetries (−0.054 and −0.009, respectively). C01 showed the largest positive asymmetries among the bilateral profiles, with values of 0.153 for SARI_core and 0.092 for SARI_ext. The corresponding values for C02 were 0.010 and 0.027.

Figure 14 shows the subject-side distribution of relative wrist height at release and peak wrist speed. A02 left was positioned among the profiles with higher values on both axes. A03 left combined a lower release height with a comparatively high peak wrist speed. The A01 left-side point showed lower values for both descriptors than the corresponding right-side point. Among the reference profiles, C01 right was positioned toward higher values of both release height and peak wrist speed.

The pre-release loading descriptors shown in Figure 15 also differed across subject-side combinations. A01 right showed greater horizontal-loading evidence than A01 left. A03 left showed greater compact-loading evidence, whereas A02 left showed substantial loading evidence within the available profile. The remaining subject-side combinations occupied different positions according to the relative contribution of horizontal and compact loading.

Taken together, Table 7 and Figure 11, Figure 12, Figure 13, Figure 14 and Figure 15 document subject- and side-specific distributions of composite SARI values, domain-level components, release height, peak wrist speed, and pre-release loading descriptors.

### 3.6. Subject-Specific Contested Shooting Profiles and Comparison with Non-Contested Shooting

**Figure 16:**
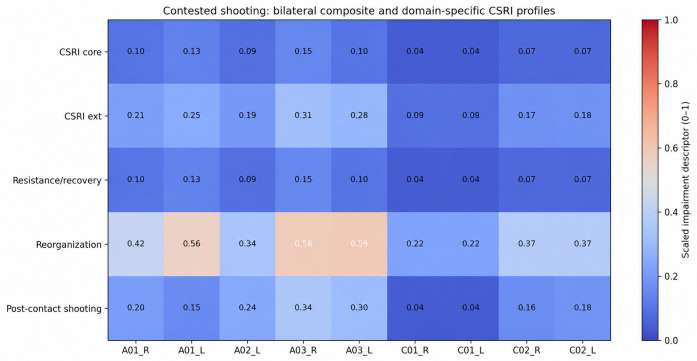
Bilateral heatmap of subject-level CSRI descriptors for the contested shooting task. Each column represents one subject-side combination, and rows report the composite indices (CSRI core and CSRI ext) together with the three domain-level descriptors: resistance/recovery, reorganization, and post-contact shooting. Higher values indicate greater task-specific functional burden according to the proposed index formulation.

**Figure 17:**
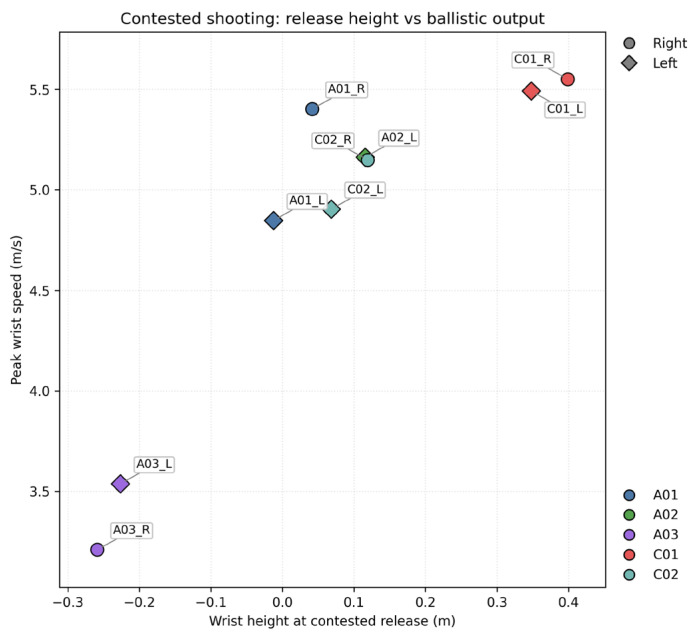
Subject-side distribution of wrist height at contested release versus peak wrist speed during the contested shooting task. Colors identify subjects and marker shapes distinguish sides. Higher values on the two axes correspond to a higher release position and greater distal output, respectively.

**Figure 18:**
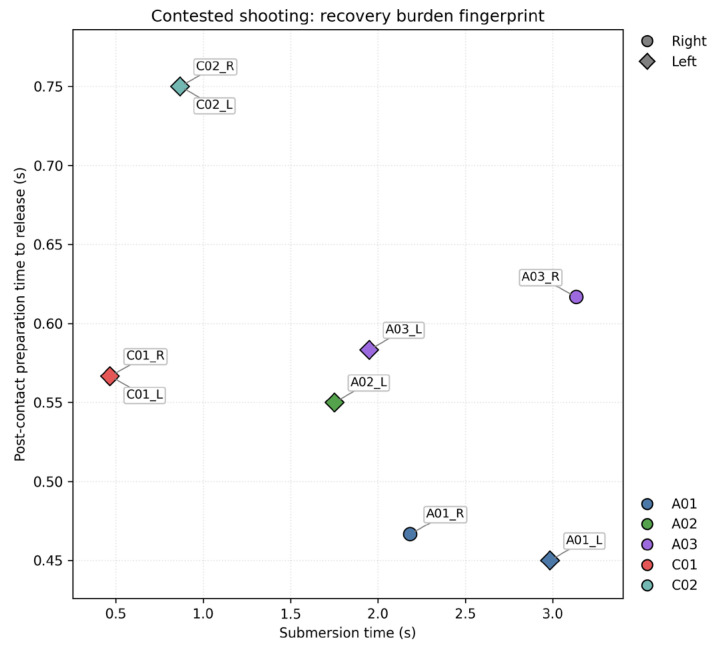
Subject-side distribution of submersion time versus post-contact preparation time to release during the contested shooting task. Colors identify subjects and marker shapes distinguish sides. Lower values on the two axes correspond to shorter submersion and post-contact preparation intervals, respectively.

**Figure 19:**
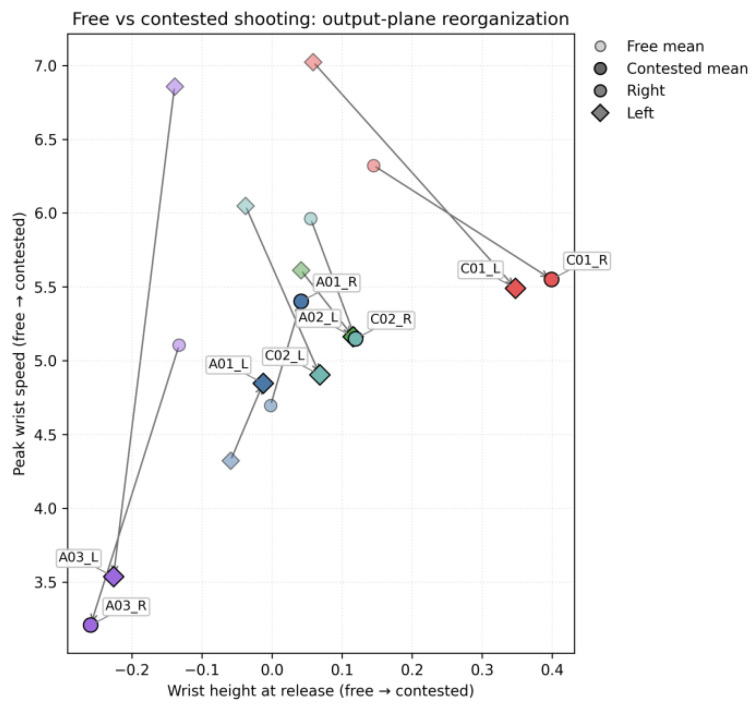
Non-contested-to-contested subject-side trajectories in the plane defined by wrist height at release and peak wrist speed. Arrows connect the non-contested condition to the contested post-contact condition for each subject-side combination and show the magnitude and direction of the change between conditions.

Subject-specific bilateral CSRI profiles for the contested shooting task are summarized in Table 8. The corresponding bilateral heatmap, release-height versus ballistic-output distribution, and recovery-burden representation are shown in Figure 16, Figure 17 and Figure 18, respectively. The within-subject, within-side comparison between non-contested and contested shooting is illustrated in Figure 19.

Across the available subject-side combinations, CSRI_core values ranged from 0.040 to 0.148, whereas CSRI_ext values ranged from 0.086 to 0.313. A01 showed higher composite values on the left than on the right, with CSRI_core values of 0.132 and 0.101 and CSRI_ext values of 0.249 and 0.209, respectively. The corresponding left-minus-right differences were 0.031 for CSRI_core and 0.040 for CSRI_ext.

A02 performed the task only with the left arm and showed CSRI_L_core and CSRI_L_ext values of 0.089 and 0.194, respectively. A03 showed CSRI_R_core and CSRI_R_ext values of 0.148 and 0.313 and corresponding left-side values of 0.099 and 0.282. This resulted in left-minus-right differences of −0.049 for CSRI_core and −0.031 for CSRI_ext.

Among the able-bodied reference participants, C01 showed CSRI_R_core and CSRI_L_core values of 0.040 on both sides, together with CSRI_R_ext and CSRI_L_ext values of 0.086 and 0.087, respectively. C02 showed CSRI_core values of 0.067 on both sides and CSRI_ext values of 0.170 on the right and 0.176 on the left. The bilateral asymmetries were therefore close to zero for both reference participants.

The domain-specific distributions shown in Figure 16 differed across subjects and sides. A03 showed the highest reorganization and post-contact-shooting values among the available profiles. A01 showed higher values on the left than on the right, whereas the available A02 left-side profile showed a lower resistance/recovery component than its post-contact-shooting component. C01 showed the lowest or near-lowest values across the three domain-level descriptors, while the corresponding C02 values were higher than those observed for C01.

Figure 17 shows the subject-side distribution of wrist height at contested release and peak wrist speed. The C01 points were located toward higher values on both axes, with the C02 points positioned below them. The A03 points showed lower release-height and peak-wrist-speed values than those of the two reference participants. The A01 bilateral points and the available A02 left-side point occupied intermediate positions within the observed distribution.

The temporal descriptors shown in Figure 18 also differed across subject-side combinations. C01 showed the shortest submersion times together with short post-contact preparation times. C02 showed longer temporal intervals than C01. A03 showed the largest combined submersion and post-contact preparation values, particularly on the right side. A01 showed comparatively long submersion times but shorter preparation intervals after re-emergence, whereas the available A02 left-side point showed comparatively short values on both axes.

Figure 19 compares wrist height at release and peak wrist speed between the non-contested and contested conditions for each available subject-side combination. The trajectories differed in both magnitude and direction across subjects and sides, and no uniform shift along either axis was observed. Some trajectories were characterized primarily by changes in release height, others by changes in peak wrist speed, and others by simultaneous changes in both descriptors.

Taken together, Table 8 and Figure 16, Figure 17, Figure 18 and Figure 19 document subject- and side-specific distributions of CSRI composite values, resistance/recovery, post-contact reorganization, shooting output, and changes between non-contested and contested shooting conditions.

## 4. Discussion

This pilot study investigated the feasibility of deriving structured, task-specific functional profiles from representative Paralympic water polo actions using stereoscopic video, markerless pose estimation, and phase-based kinematic analysis. Overall, the proposed framework allowed subject-, side-, and task-specific differences to be described under conditions characterized by partial observability, water-surface interference, submersion, and physical opposition. The exact frame-level agreement between automatic and manual ball-release identification across the analyzed ball-related trials also supported the use of release-centered segmentation within the present dataset.

The different tasks provided complementary information on the functional organization of performance. Upright floating showed relatively limited variation in the global composite indices, while revealing different distributions across postural stability, head–trunk coordination, upper-body compensation, and buoyancy-maintenance components. In the present cohort, this task therefore appeared to be more informative for describing the organization of short-window postural control than for identifying large differences in global composite values.

Vertical propulsion produced a wider distribution of subject-level profiles. In the combined arm-and-leg condition, differences involved not only the propulsion-core descriptors but also temporal stability, alignment control, upper-body contribution, and post-thrust recovery. The non-identical distributions of these components suggest that similar global index values may reflect different combinations of propulsion generation and stabilization demands. In the legs-only condition, the propulsion-core component showed the widest between-subject range, whereas alignment-control and upper-body-strategy values varied within narrower intervals. This pattern is consistent with the stronger constraint placed on lower-body-generated vertical propulsion when upper-limb contribution is restricted.

Passing and non-contested shooting extended the analysis to unilateral upper-limb function. The bilateral profiles documented side-specific differences in composite indices, release organization, distal output, proximal–distal coordination, postural control, and trunk involvement. Importantly, the direction and magnitude of asymmetry were not uniform across participants or tasks. These findings support the use of side-specific profiling rather than reliance on a single whole-body score, particularly when residual function and compensatory strategies may differ between limbs.

Contested shooting provided additional information that was not fully represented by the non-contested condition. The phase-based decomposition into pre-contact, submersion, re-emergence, and post-contact execution enabled perturbation tolerance and motor reorganization to be examined without reconstructing artificial trajectories through periods of invalid visibility. The comparison between non-contested and contested shooting did not show a uniform change in release height or peak wrist speed across subject-side combinations. Instead, the trajectories differed in magnitude and direction, indicating subject-specific combinations of post-contact recovery, release geometry, distal output, and trunk contribution. Accordingly, the contested condition should not be interpreted simply as producing a generalized degradation score. It provides a complementary description of how shooting execution is reorganized under physical opposition.

A central methodological aspect of the proposed framework is the explicit treatment of temporary visibility loss. Markerless motion-analysis methods commonly regard tracking interruptions as missing data to be reconstructed or discarded. In the present aquatic setting, however, submersion and re-emergence may constitute intrinsic components of task execution. By defining descriptors only within valid, temporally contiguous phases and excluding unavailable KeyPoints without interpolation, the framework preserves the physical meaning of the observed trajectories. At the same time, this approach means that descriptor availability depends on the visibility and continuity criteria reported in the Supplementary Materials.

The findings must be interpreted in light of the exploratory design. First, the cohort was small and intentionally heterogeneous. The two able-bodied participants were included as reference profiles only and do not constitute a normative control group. Consequently, the observed distributions cannot establish classification thresholds or demonstrate formal sensitivity or discriminability between functional groups. Second, although multiple trials were acquired within the experimental session, repeated-session test–retest reliability was not assessed. Third, the proposed indices were not compared with expert classification judgments or independent functional benchmarks. They should therefore be considered candidate functional descriptors rather than validated classification measures.

A further limitation concerns the technical validation of the measurement pipeline. Although the adopted pose-estimation and stereo-reconstruction approaches are grounded in established computer-vision methods [6,7,8,9,10,11] and previous studies have characterized both the feasibility and the limitations of markerless kinematic analysis in laboratory and sport-specific settings [12,13,14,15,16,22,23], their measurement error was not quantified against a gold-standard motion-capture system under the specific aquatic conditions examined here. Reviews of markerless motion capture emphasize that accuracy remains dependent on the task, environment, camera setup, and biomechanical variable considered [20], while underwater pose-estimation research identifies turbulence, bubbles, and other aquatic disturbances as additional challenges [21]. These considerations are especially relevant here, because water reflections, splashes, partial submersion, and short periods of reduced KeyPoint visibility may affect reconstruction precision. The present results consequently support methodological feasibility and descriptive profiling, but not yet full measurement validity.

Future studies should involve larger and more diverse cohorts, repeated acquisition sessions, formal reliability and measurement-error analyses, and comparison with expert technical and classification assessments. Quantitative evaluation of sensitivity and discriminability will also require adequately powered samples representing different impairment and functional profiles. These validation steps will be necessary before the proposed indices can be considered for practical integration into classification-oriented assessment.

## 5. Conclusions

This pilot study presents a task-specific framework for deriving interpretable kinematic profiles from ecologically relevant Paralympic water polo actions. The combination of stereoscopic video, markerless three-dimensional reconstruction, and phase-based analysis enabled postural control, vertical propulsion, unilateral upper-limb function, and post-contact motor reorganization to be described at subject and side level.

The proposed approach also enabled visibility interruptions and submersion-related discontinuities to be incorporated into the functional organization of the analysis rather than reconstructed through interpolation. The resulting descriptors and composite indices are not intended to replace official classification procedures or to automate Sport Class assignment. They should instead be regarded as exploratory candidate measures providing a methodological basis for future studies of reliability, measurement validity, discriminability, and alignment with expert classification practice.

## Figures and Tables

**Table 1:** Individual characteristics of the study participants.

Subject	Role in the Study	Age (Years)	Height (cm)	Weight (kg)	Eligible Impairment/Functional Condition	Main Affected Side or Body Region	Official Sport, if Available ^1^
A01	Athlete with eligible impairment	26	172	70	Permanent unilateral upper-limb impairment	Left upper limb	Not available
A02	Athlete with eligible impairment	36	175	78	Permanent unilateral upper-limb impairment	Right upper limb	Not available
A03	Athlete with eligible impairment	28	174	95	Permanent bilateral lower-limb impairment	Lower limbs	Not available
C01	Able-bodied reference participant	33	185	88	None	Not applicable	Not applicable
C02	Able-bodied reference participant	65	179	83	None	Not applicable	Not applicable

^1^ Official Sport Class information was not available for the present study. The able-bodied participants were included as reference profiles only and were not used to establish normative values.

**Table 2:** Summary of tasks, functional domains, feature tiers, and derived indices in the proposed framework.

Task	Main Functional Domain (s)	Phase Structure/ Analysis Window	Feature Tiers Used	Derived Index/ Main Output
Upright Floating	Postural control; buoyancy management; stabilization strategies	Short stable pre-propulsion window extracted from dynamic tasks	Tier 1–3	Static Floating Index (SFI)
Explosive Vertical Propulsion	Vertical propulsion capacity; trunk stabilization; segmental contribution	Event-centered propulsion window including pre-thrust, thrust, and post-thrust stabilization	Tier 1–3	Vertical Propulsion Index for combined condition (VPI_LA); Vertical Propulsion Index for legs-only condition (VPI_L)
Passing	Unilateral upper-limb propulsion; release organization; proximal–distal coordination; compensatory trunk involvement	Release-centered passing windows including pre-release, release-centered, and post-release/recovery phases	Tier 1–3	Passing Arm Residual Index (PARI_R, PARI_L); inter-limb comparison descriptors
Non-Contested Shooting	Explosive upper-limb propulsion; release geometry; coordination under stable floating conditions; postural control under load	Release-centered shooting windows including pre-release, release-centered, and post-release/recovery phases	Tier 1–3	Shooting Arm Residual Index (SARI_R, SARI_L)
Contested Shooting under Physical Opposition	Perturbation tolerance; post-contact recovery; residual shooting capacity under constraint; post-contact motor reorganization	Phase-based segmentation: pre-contact, contact/submersion, and post-contact phases; automatically detected release used to refine post-contact execution and cross-condition comparison when available	Tier 1–4	Contested Shooting Resistance Index (CSRI_R, CSRI_L); derived integrated descriptors; non-contested vs. contested comparison descriptors

**Table 3:** Subject-level composite and domain-specific SFI descriptors during Upright Floating.

Subject	Role in Study	SFI Core	SFI Ext	Postural Core	Head–Trunk Coordination	Shoulder Compensation	Maintenance Effort
A01	Eligible impairment profile	0.14	0.14	0.17	0.08	0.12	0.24
A02	Eligible impairment profile	0.15	0.16	0.11	0.27	0.13	0.24
A03	Eligible impairment profile	0.10	0.12	0.10	0.09	0.05	0.24
C01	Reference athlete profile	0.05	0.10	0.03	0.11	0.10	0.18
C02	Reference athlete profile	0.11	0.09	0.06	0.26	0.03	0.15

**Table 4:** Subject-level composite and domain-specific VPI_LA descriptors during Explosive Vertical Propulsion (legs + arms).

Subject	VPI_LA Core	VPI_LA Ext	Propulsion Core	Temporal Stability	Alignment Control	Upper-Body Strategy	Retention/Recovery
A01	0.49	0.40	0.46	0.33	0.36	0.55	0.57
A02	0.53	0.41	0.80	0.45	0.13	0.22	0.47
A03	0.68	0.50	0.91	0.52	0.42	0.29	0.39
C01	0.29	0.28	0.26	0.26	0.14	0.19	0.56
C02	0.49	0.42	0.78	0.46	0.07	0.21	0.60

**Table 5:** Subject-level VPI_L profiles for the explosive vertical propulsion task performed with legs only. Higher values indicate greater task-specific functional burden according to the proposed index formulation.

Subject	VPI_L Core	VPI_L Ext	Propulsion Core	Temporal Stability	Alignment Control	Upper-Body Strategy	Retention/Recovery
A01	0.43	0.33	0.70	0.20	0.08	0.20	0.53
A02	0.26	0.22	0.38	0.33	0.08	0.22	0.10
A03	0.59	0.36	0.86	0.24	0.22	0.23	0.30
C01	0.24	0.22	0.36	0.32	0.08	0.16	0.18
C02	0.29	0.29	0.44	0.29	0.12	0.17	0.44

**Table 6:** Subject-level bilateral PARI profiles for the ball passing task. Right and left arm indices are reported separately, and asymmetry is expressed as left-minus-right difference (L−R). Higher values indicate greater task-specific functional burden.

Subject	PARI_R Core	PARI_R Ext	PARI_L Core	PARI_L Ext	PARI_ASYM Core (L−R)	PARI_ASYM Ext (L−R)
A01	0.308	0.308	0.557	0.509	0.249	0.200
A02	–	–	0.277	0.398	–	–
A03	0.819	0.668	0.713	0.588	−0.106	−0.079
C01	0.167	0.181	0.229	0.270	0.063	0.089
C02	0.378	0.347	0.456	0.344	0.078	−0.002

**Table 7:** Subject-level bilateral SARI profiles for the ball shooting task. Right and left arm indices are reported separately, and asymmetry is expressed as left-minus-right difference (L−R). Higher values indicate greater task-specific functional burden according to the proposed index formulation.

Subject	SARI_R Core	SARI_R Ext	SARI_L Core	SARI_L Ext	SARI_ASYM Core (L−R)	SARI_ASYM Ext (L−R)
A01	0.232	0.202	0.282	0.270	0.050	0.068
A02	—	—	0.219	0.264	—	—
A03	0.290	0.241	0.235	0.232	−0.054	−0.009
C01	0.122	0.112	0.275	0.204	0.153	0.092
C02	0.296	0.197	0.306	0.224	0.010	0.027

**Table 8:** Subject-level bilateral CSRI profiles for the contested shooting task. Right and left arm indices are reported separately, and asymmetry is expressed as the left-minus-right difference (L−R). Higher values indicate greater task-specific functional burden according to the proposed index formulation.

Subject	CSRI_R Core	CSRI_R Ext	CSRI_L Core	CSRI_L Ext	CSRI_ASYM Core (L−R)	CSRI_ASYM Ext (L−R)
A01	0.101	0.209	0.132	0.249	0.031	0.040
A02	–	–	0.089	0.194	–	–
A03	0.148	0.313	0.099	0.282	−0.049	−0.031
C01	0.040	0.086	0.040	0.087	0.000	0.001
C02	0.067	0.170	0.067	0.176	0.000	0.006

## Data Availability

Data not publicly available due to privacy/ethical restrictions.:

## Supplementary Materials

The following supporting information can be downloaded at: https://www.mdpi.com/article/10.3390/bioengineering13060707/s1, Figure S1: High-level workflow of the proposed functional profiling framework; Table S1: Effective trial availability by subject, task, and side; Table S2: KeyPoint validity criteria and operational phase/event definitions; Table S3: Acquisition, reconstruction, and trajectory-processing parameters; Table S4: Feature-level scaling rules and composite-index composition; Table S5: Upright Floating—Tier 1 Core Postural Control Descriptors; Table S6: Upright Floating—Tier 2 Upper-Body Compensatory Strategy Descriptors; Table S7: Upright Floating—Tier 3 Buoyancy-Maintenance Proxy Descriptors; Table S8: Explosive Vertical Propulsion from Upright Floating—Tier 1 Core Propulsion and Stabilization Descriptors; Table S9: Explosive Vertical Propulsion from Upright Floating—Tier 2 Upper-Limb Contribution Descriptors; Table S10: Explosive Vertical Propulsion from Upright Floating—Tier 3 Post-Thrust Stabilization and Lower-Limb Contribution Descriptors; Table S11: Passing—Tier 1 Postural Control Descriptors; Table S12: Passing—Tier 2 Active-Arm Kinematic and Coordination Descriptors; Table S13: Passing—Tier 3 Compensatory Strategy Descriptors; Table S14: Passing—Additional Inter-Limb Comparison Descriptors; Table S15: Non-Contested Shooting—Tier 1 Postural Control Descriptors; Table S16: Non-Contested Shooting—Tier 2 Upper-Limb Explosive Capacity, Release Geometry, and Loading Descriptors; Table S17: Non-Contested Shooting—Tier 3 Compensatory Strategy and Propulsion-Origin Descriptors; Table S18: Contested Shooting under Physical Opposition—Tier 1 Resistance and Recovery Descriptors; Table S19: Contested Shooting under Physical Opposition—Tier 2 Post-Contact Motor Reorganization Descriptors; Table S20: Contested Shooting under Physical Opposition—Tier 3 Post-Contact Shooting Descriptors; Table S21: Contested Shooting under Physical Opposition—Tier 4 Derived Integrated Descriptors; Table S22: Descriptor set for within-subject, within-arm comparison between non-contested shooting and post-contact contested shooting.
