# Supplementary material for: Functional Profiling in Paralympic Water Polo Using Deep Learning, Stereo Vision, and Phase-Based Kinematic Analysis: A Pilot Study
Source: Bioengineering (Basel). 2026 Jun 19;13(6):707. doi: 10.3390/bioengineering13060707 (PMC13295668; doi:10.3390/bioengineering13060707)
Supplement: Supplementary file 1 [file bioengineering-13-00707-s001.zip › bioengineering-4333484-supplementary.pdf]

Figure S1: High-level workflow of the proposed functional profiling framework

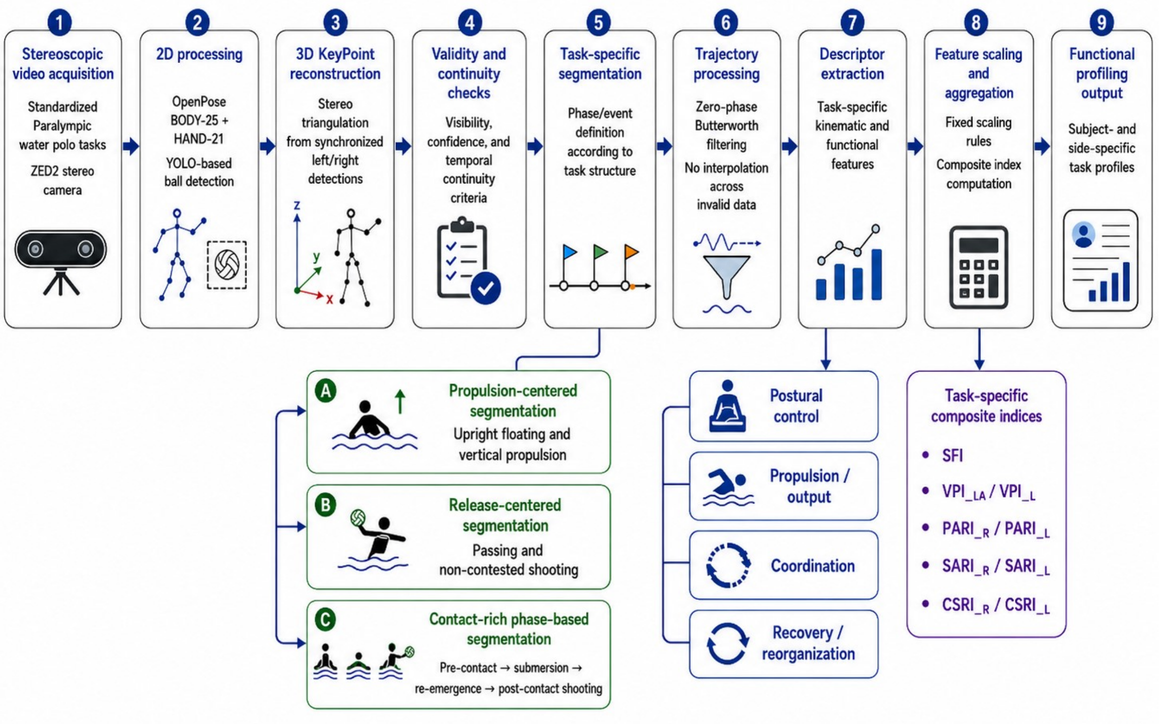

Figure S1. High-level workflow of the proposed functional profiling framework. Stereoscopic video recordings were processed through 2D pose estimation and ball detection, 3D KeyPoint reconstruction, validity and continuity checks, task-specific phase or event segmentation, trajectory filtering, descriptor extraction, and feature scaling and aggregation. Three segmentation strategies were adopted according to task structure: propulsion-centered segmentation for upright floating and vertical propulsion, release-centered segmentation for passing and non-contested shooting, and contact-rich phase-based segmentation for contested shooting. The resulting descriptors were aggregated into task-specific indices to generate subject- and side-specific functional profiles.

Table S1. Effective trial availability by subject, task, and side.

Subject	Task	Side / condition	Acquired trials	Retained trials	Excluded trials	Main reason for exclusion
A01	Upright floating	—	6	6	0	N/A
A01	Vertical propulsion	Arms + legs	6	6	0	N/A
A01	Vertical propulsion	Legs only	6	6	0	N/A
A01	Passing	Right	6	6	0	N/A
A01	Passing	Left	6	6	0	N/A
A01	Non-contested shooting	Right	6	6	0	N/A
A01	Non-contested shooting	Left	6	6	0	N/A
A01	Contested shooting	Right	6	6	0	N/A
A01	Contested shooting	Left	6	5	1	partial loss of trunk/head visibility during the target phase
A02	Upright floating	—	6	6	0	N/A
A02	Vertical propulsion	Arms + legs	6	6	0	N/A
A02	Vertical propulsion	Legs only	6	6	0	N/A
A02	Passing	Right	—	—	—	not applicable (task not performed on this side)
A02	Passing	Left	6	6	0	N/A
A02	Non-contested shooting	Right	—	—	—	not applicable (task not performed on this side)
A02	Non-contested shooting	Left	6	4	2	water-splash occlusion affecting upper-limb visibility
A02	Contested shooting	Right	—	—	—	not applicable (task not performed on this side)
A02	Contested shooting	Left	6	4	2	water-splash occlusion affecting upper-limb visibility
A03	Upright floating	—	6	6	0	N/A
A03	Vertical propulsion	Arms + legs	6	6	0	N/A

Subject	Task	Side / condition	Acquired trials	Retained trials	Excluded trials	Main reason for exclusion
A03	Vertical propulsion	Legs only	6	4	2	partial loss of trunk/head visibility during the target phase
A03	Passing	Right	6	4	2	partial loss of trunk/head visibility during the target phase
A03	Passing	Left	6	5	1	partial loss of trunk/head visibility during the target phase
A03	Non-contested shooting	Right	6	4	2	partial loss of trunk/head visibility during the target phase
A03	Non-contested shooting	Left	6	4	2	partial loss of trunk/head visibility during the target phase
A03	Contested shooting	Right	6	4	2	partial loss of trunk/head visibility during the target phase
A03	Contested shooting	Left	6	4	2	partial loss of trunk/head visibility during the target phase
C01	Upright floating	—	6	6	0	N/A
C01	Vertical propulsion	Arms + legs	6	6	0	N/A
C01	Vertical propulsion	Legs only	6	6	0	N/A
C01	Passing	Right	6	4	2	water-splash occlusion affecting upper-limb visibility
C01	Passing	Left	6	4	2	water-splash occlusion affecting upper-limb visibility
C01	Non-contested shooting	Right	6	4	2	water-splash occlusion affecting upper-limb visibility
C01	Non-contested shooting	Left	6	4	2	water-splash occlusion affecting upper-limb visibility
C01	Contested shooting	Right	6	4	2	water-splash occlusion affecting upper-limb visibility
C01	Contested shooting	Left	6	4	2	water-splash occlusion affecting upper-limb visibility
C02	Upright floating	—	6	6	0	N/A
C02	Vertical propulsion	Arms + legs	6	6	0	N/A
C02	Vertical propulsion	Legs only	6	6	0	N/A

Subject	Task	Side / condition	Acquired trials	Retained trials	Excluded trials	Main reason for exclusion
C02	Passing	Right	6	6	0	N/A
C02	Passing	Left	6	6	0	N/A
C02	Non-contested shooting	Right	6	4	2	water-splash occlusion affecting upper-limb visibility
C02	Non-contested shooting	Left	6	5	1	water-splash occlusion affecting upper-limb visibility
C02	Contested shooting	Right	6	4	2	water-splash occlusion affecting upper-limb visibility
C02	Contested shooting	Left	6	5	1	water-splash occlusion affecting upper-limb visibility

Table S2. KeyPoint validity criteria and operational phase/event definitions

A. KeyPoint validity criteria

Criterion	Operational definition	Rule adopted
2D KeyPoint confidence	Minimum confidence required for a 2D KeyPoint detection to be considered valid in a given view	≥ 0.15
Stereo availability for 3D reconstruction	3D coordinates reconstructed only when the corresponding KeyPoint was valid in both left and right views at the same frame	Required
Window-level KeyPoint validity	A KeyPoint was considered usable within an analysis window only when it remained valid for a sufficient portion of the window	≥ 70% of frames
Minimum contiguous segment length	Minimum number of consecutive valid frames required for phase-based descriptor computation	≥ 5 consecutive frames
Maximum tolerated interruption	Very short interruptions tolerated within an otherwise valid segment	≤ 2 consecutive frames
Interpolation across invalid frames	Missing or unreliable KeyPoints were not reconstructed by interpolation	Not allowed
Descriptor exclusion rule	A descriptor was set to missing when one or more required KeyPoints did not satisfy the validity criteria within the relevant window	Applied at descriptor level
Trial retention logic	A trial was retained when the task event/phase anchor was valid and at least a subset of core descriptors could be computed	Descriptor-level retention; no full-trial imputation

B. Operational phase and event definitions

Event / boundary	Operational definition
Upright-floating analysis window	Short pre-propulsion interval with minimal voluntary motion, selected by minimizing vertical velocity and acceleration of the neck KeyPoint
Propulsion onset (vertical propulsion)	First frame in which the neck vertical velocity exceeded a task-specific threshold after a stable pre-thrust interval
Propulsion end (vertical propulsion)	Frame corresponding to peak vertical displacement reached after the upward thrust
Ball release (passing / non-contested shooting)	First frame in which the ball was no longer in contact with the active hand and began an independent trajectory, as supported by ball localization and distal upper-limb KeyPoints
Manual verification	Automatic release timing was manually checked at frame level in all analyzed ball-related trials (n = 128), showing exact agreement in all cases.
Pre-release window	Fixed window preceding the automatically detected ball-release event
Release-centered window	Fixed window centered on the automatically detected ball-release event
Post-release / recovery window	Fixed window following the automatically detected ball-release event
Contact onset (contested shooting)	Last valid frame preceding sustained upper-body visibility loss
Re-emergence (contested shooting)	First frame with restored upper-body visibility after the contact/submersion phase
Post-contact release (contested shooting)	Automatically detected release occurring after re-emergence and within a temporally contiguous post-contact execution segment

Table S3. Acquisition, reconstruction, and trajectory-processing parameters

Parameter	Setting adopted in the present study
Camera system	ZED2 stereo camera (Stereolabs)
Stereo baseline	0.12 m
Acquisition rate	60 Hz
Image resolution	2560 × 720 pixels total (1280 × 720 per sensor)
Camera placement	Fixed across participants and trials
Stereo calibration	Manufacturer-provided recalibration procedure
Additional custom spatial calibration	None
3D reconstruction input	Synchronized left/right 2D KeyPoint detections
3D reconstruction method	Stereo triangulation using calibrated stereo parameters
Pose-estimation model	OpenPose v1.5.1

Parameter	Setting adopted in the present study
Body model	BODY-25
Hand model	HAND-21
Ball detection	YOLO-based detector
Global coordinate system	Native ZED reference frame
Axis convention	Right-handed frame: X to the right, Y downward, Z forward from the camera
Additional coordinate transformations	None
Temporal filtering	Fourth-order low-pass Butterworth filter
Filter implementation	Zero-phase, applied independently to each spatial component
Cut-off frequency — upright floating	3 Hz
Cut-off frequency — explosive vertical propulsion	5 Hz
Cut-off frequency — passing	6 Hz
Cut-off frequency — non-contested shooting	6 Hz
Cut-off frequency — contested shooting	5 Hz
Interpolation of missing trajectories	Not applied
Feature computation	Based on filtered trajectories only

Table S4. Feature-level scaling rules and composite-index composition

A. General scaling logic

Descriptor family	Directionality after alignment	Proposed scaling rule
Variability / sway / jerk measures	Higher raw values indicate poorer control	Direct bounded scaling to [0,1]
Time-to-event / delay / prolonged-duration measures	Higher raw values indicate poorer performance	Direct bounded scaling to [0,1]
Peak velocity / acceleration / impulse / displacement / release height	Lower raw values indicate poorer performance	Inverse bounded scaling to [0,1]
Consistency / stability / coupling measures	Lower raw values indicate poorer performance	Inverse bounded scaling to [0,1]
Asymmetry measures	Larger absolute deviation indicates poorer performance	Direct bounded scaling of absolute value
Binary completion / success descriptors	Successful execution more favorable	Success = 0; failure / incomplete = 1

Descriptor family	Directionality after alignment	Proposed scaling rule
Recovery-burden descriptors	Higher burden indicates poorer performance	Direct bounded scaling to [0,1]

B. Composite-index composition

Index	Core formulation	Extended formulation	Weighting rule
SFI	Reduced subset of descriptors from Table S5 capturing vertical steadiness, horizontal sway, alignment stability, and corrective smoothness	Full descriptor set from Tables S5–S7	Equal weighting
VPI_LA	Robust propulsion and stabilization descriptors from Table S8	Full descriptor set from Tables S8–S10	Equal weighting
VPI_L	Legs-only applicable propulsion and stabilization descriptors from Table S8 and stabilization descriptors from Table S10	Full legs-only applicable descriptor set from Tables S8 and S10	Equal weighting
PARI_R / PARI_L	Reduced subset of descriptors from Tables S11–S12 emphasizing postural control, release-centered arm output, and coordination	Full descriptor set from Tables S11–S13	Equal weighting
SARI_R / SARI_L	Reduced subset of descriptors from Tables S15–S16 emphasizing ballistic output, release geometry, and upper-limb coordination	Full descriptor set from Tables S15–S17	Output-weighted composite, with greater emphasis on ballistic execution and release configuration
CSRI_R / CSRI_L	Robust event- and outcome-based descriptors from Tables S18–S19	Full descriptor set from Tables S18–S20	Equal weighting
Tier 4 contested-shooting descriptors	Not included in CSRI computation	Reported separately in Table S21	Not aggregated into CSRI
Within-subject non-contested vs contested comparison descriptors	Not included in composite indices	Reported separately in Table S22	Comparative only

Table S5. Upright Floating — Tier 1 Core Postural Control Descriptors

Feature	Description	Functional interpretation

Feature	Description	Functional interpretation
Vertical level variability	Standard deviation of vertical position during the static window (m)	Ability to maintain a steady floating level (core postural control)
Horizontal sway (XY)	RMS horizontal displacement during the static window (m)	Global stability in the horizontal plane; reduced sway indicates better balance control
Lateral sway (X)	RMS medio-lateral displacement during the static window (m)	Side-to-side stability and symmetry of balance reactions
Depth sway (Y)	RMS antero-posterior displacement during the static window (m)	Forward–backward balance control and alignment maintenance
Vertical velocity variability	Standard deviation of vertical velocity during the static window (m/s)	Amount of vertical corrective action while maintaining buoyancy
Vertical smoothness (jerk)	RMS of vertical jerk during the static window (m/s^3)	Neuromotor smoothness of postural corrections; higher values indicate abrupt control actions
Upper-body alignment variability	Standard deviation of head–neck alignment angle relative to vertical (deg)	Stability of upright posture; reflects control of body orientation in water
Head–trunk vertical coupling	Cross-correlation of head and trunk vertical velocities in the static window (-)	Intersegmental coordination during small postural corrections
Head lag	Temporal delay between head and trunk vertical motion in the static window (s)	Timing of segmental stabilization; delays suggest impaired proximal–distal control

Table S6. Upright Floating — Tier 2 Upper-Body Compensatory Strategy Descriptors

Feature	Description	Functional interpretation
Shoulder asymmetry (vertical)	RMS difference between left and right shoulder vertical positions in the static window (m)	Asymmetric upper-limb posture/control; may reflect unilateral impairment or compensatory stabilization
Upper-body counter-rotation variability	Standard deviation of shoulder-line orientation relative to the head–neck axis (deg)	Trunk–shoulder dissociation used as a stabilizing strategy; elevated variability may indicate compensation
Shoulder sway (XY)	RMS horizontal displacement of the shoulder midpoint in the static window (m)	Upper-limb contribution to balance reactions (when available)
Shoulder sway asymmetry	Difference in horizontal sway magnitude between left and right shoulders (m)	Uneven stabilizing contribution; may reveal compensatory redistribution across upper limbs

Table S7. Upright Floating — Tier 3 Buoyancy-Maintenance Proxy Descriptors

Feature	Description	Functional interpretation
Vertical drift	Difference between final and initial vertical position within the static window (m)	Tendency to sink/rise over a short period; proxy of buoyancy maintenance effectiveness
Stabilization consistency	Inverse normalized variance of vertical position in the static window (-)	Reliability of maintaining a stable level; higher values indicate more consistent control
Control effort proxy (vertical)	RMS of vertical acceleration magnitude in the static window (m/s^2)	Amount of corrective effort required to maintain floating; higher effort suggests reduced efficiency
Sway-to-level ratio	Ratio of horizontal sway RMS to vertical variability (-)	Trade-off between horizontal corrections and vertical steadiness; reflects control efficiency under constraint

Table S8. Explosive Vertical Propulsion from Upright Floating — Tier 1 Core Propulsion and Stabilization Descriptors

Feature	Description	Functional interpretation
Thrust onset time	Time of propulsion onset (s)	Initiation of voluntary upward impulse
Thrust offset time	End of positive propulsion phase (s)	Termination of effective propulsion
Peak time	Time of maximum upward velocity (s)	Reference instant for post-impulse control
Thrust duration	Duration of propulsion phase (s)	Efficiency vs prolonged propulsion strategy
Time to peak velocity	Time from onset to velocity peak (s)	Rapidity of motor execution
Peak vertical velocity	Maximum upward vertical velocity (m/s)	Whole-body upward momentum generation
Peak vertical acceleration	Maximum vertical acceleration near onset (m/s^2)	Rate of force development
Positive vertical impulse	Integral of positive vertical velocity (m)	Global effectiveness of propulsion
Net vertical displacement (thrust)	Vertical position change during propulsion (m)	Immediate task outcome (elevation gain/loss)
Propulsion efficiency (per time)	Vertical displacement normalized by thrust duration (-)	Mechanical effectiveness of propulsion
Propulsion efficiency (per impulse)	Vertical displacement normalized by vertical impulse (-)	Conversion of impulse into maintained elevation
Pre-thrust vertical stability	Standard deviation of vertical position before onset (m)	Quality of postural preparation
Post-thrust sway (XY)	RMS horizontal displacement after peak (m)	Dynamic balance recovery after propulsion
Post-stabilization time	Time required to regain steady floating after thrust (s)	Ability to stabilize body posture
Upper-body alignment angle	Angle between head–neck axis and vertical (deg)	Upright postural control affecting hydrodynamic efficiency
Lateral deviation RMS	RMS medio-lateral displacement (m)	Directional stability
Depth deviation RMS	RMS antero-posterior displacement (m)	Forward–backward alignment control
Vertical jerk RMS	RMS of vertical jerk during propulsion and stabilization (m/s^3)	Smoothness of motion and coordination
Head–trunk vertical coupling	Cross-correlation of head and trunk vertical velocities (-)	Intersegmental coordination efficiency
Head lag	Temporal delay between head and trunk motion (s)	Proximal–distal timing during stabilization
Explosive-to-stabilization ratio	Ratio between propulsion and post-thrust sway metrics (-)	Trade-off between thrust generation and control
Double thrust flag	Presence of secondary corrective impulse (-)	Compensatory adjustment during propulsion

Table S9. Explosive Vertical Propulsion from Upright Floating — Tier 2 Upper-Limb Contribution Descriptors

Feature	Description	Functional interpretation
Upper-body counter-rotation	Variability of shoulder orientation relative to the head–neck axis (deg)	Compensatory trunk–shoulder dissociation during propulsion
Propulsion asymmetry (shoulders)	Difference in vertical displacement between left and right shoulders during thrust (m)	Uneven upper-limb contribution to propulsion
Shoulder vertical range of motion	Peak-to-peak vertical displacement of shoulders during thrust (m)	Extent of upper-limb involvement in impulse generation
Shoulder–trunk coordination	Cross-correlation of shoulder and trunk vertical velocities (-)	Intersegmental coordination between upper limbs and core
Shoulder lag	Temporal delay between shoulder and trunk motion (s)	Timing of upper-limb contribution during stabilization

Table S10. Explosive Vertical Propulsion from Upright Floating — Tier 3 Post-Thrust Stabilization and Lower-Limb Contribution Descriptors

Feature	Description	Functional interpretation
Elevation retention	Fraction of vertical elevation maintained after propulsion (-)	Ability to sustain upward displacement following impulse
Post-peak elevation loss	Vertical displacement loss from peak to stabilization (m)	Energy dissipation due to insufficient postural control
Stabilization velocity decay	Rate of reduction of vertical velocity after peak (m/s^2)	Effectiveness of post-impulse damping
Time to neutral velocity	Time from peak to zero vertical velocity (s)	Ability to arrest upward motion and stabilize posture
Post-thrust vertical drift	Vertical displacement during stabilization phase (m)	Maintenance of floating level after propulsion
Secondary descent amplitude	Maximum downward displacement after peak (m)	Instability following propulsion
Stabilization consistency	Variability of vertical position during steady floating (-)	Reliability of postural maintenance

General note for all ball-related tasks

For all ball-related tasks, the central temporal reference is the automatically detected ball release, obtained by combining YOLO-based ball localization with the frame-wise spatial relationship between the ball and the active hand. Feature extraction is performed using fixed windows referenced to this event rather than variable onset–offset intervals derived only from wrist-speed thresholds. Unless otherwise specified, three temporal regions are considered: a pre-release window, a release-centered window, and a post-release/recovery window. This approach reduces dependence on variable preparation duration, follow-through duration, and partial occlusion, while improving biomechanical interpretability across trials and subjects.

Table S11. Passing — Tier 1 Postural Control Descriptors

Feature	Description (unit)	Functional interpretation
Ball release time	Timestamp of automatically detected ball release (s)	Reference time-point for time-locking the passing gesture
Trunk horizontal sway at release	RMS horizontal displacement of the trunk reference during a short release-centered window (m)	Postural stability at the crucial instant of ball release
Trunk vertical level variability pre-release	Standard deviation of trunk vertical position during the fixed pre-release window (m)	Ability to maintain buoyant level while preparing the pass
Preparatory trunk stability	RMS 3D trunk speed during the fixed pre-release window (m/s)	Quality of stabilization/readiness before force transmission
Post-pass recovery sway	RMS horizontal displacement of the trunk reference during the fixed post-release window (m)	Ability to rapidly regain steadiness after the pass
Postural cost of passing	Ratio between release-centered trunk horizontal sway and peak wrist speed in the pre-release window (-)	Stability disturbance required to generate distal propulsion

Table S12. Passing — Tier 2 Active-Arm Kinematic and Coordination Descriptors

Feature	Description (unit)	Functional interpretation
Peak wrist speed pre-release	Maximum 3D wrist speed relative to the trunk during the fixed pre-release window (m/s)	Throwing intensity / capacity to generate terminal hand speed before release
Peak wrist acceleration pre-release	Maximum wrist acceleration during the fixed pre-release window (m/s^2)	Explosiveness / rate of force development (“snap”)
Wrist path length pre-release	Total 3D wrist displacement during the fixed pre-release window (m)	Movement amplitude and economy of the effective passing phase
Release-to-peak wrist timing	Time difference between ball release and peak wrist speed (s)	Temporal organization of force transmission relative to release
Elbow vertical range of motion pre-release	Peak-to-peak elbow vertical displacement relative to the trunk during the pre-release window (m)	Contribution of elbow drive/elevation to the throw
Elbow–wrist coordination pre-release	Maximum cross-correlation between elbow and wrist speed profiles during the pre-release window (-)	Proximal–distal coordination quality (kinetic chain efficiency)
Proximal–distal lag pre-release	Time lag at maximum elbow–wrist speed correlation during the pre-release window (s)	Sequencing of elbow vs wrist contribution (timing strategy)
Wrist smoothness pre-release	RMS jerk-related derivative of wrist speed during the pre-release window (m/s^3)	Coordination quality: higher values indicate abrupt or compensatory control
Wrist path efficiency pre-release	Ratio between straight-line wrist displacement and total trajectory length during the pre-release window (-)	Movement economy: lower values indicate corrective motion
Pre-release fragmentation index	Number of secondary wrist-speed peaks preceding the main pre-release peak (-)	Structural organization of the passing gesture

Table S13. Passing — Tier 3 Compensatory Strategy Descriptors

Feature	Description (unit)	Functional interpretation
Trunk rotation variability pre-release	Standard deviation of shoulder-line orientation in the horizontal plane during the pre-release window (deg)	Use of trunk rotation as compensatory strategy to support the pass
Shoulder asymmetry at release	Absolute vertical difference between left and right shoulders at ball release (m)	Unilateral stabilization strategy / asymmetric upper-body involvement at the release instant
Relative wrist propulsion index pre-release	Ratio between wrist displacement relative to the trunk and trunk displacement during the pre-release window (-)	Arm-driven propulsion vs compensatory trunk motion
Torso displacement pre-release	RMS displacement of the trunk reference during the pre-release window (m)	Degree of whole-body involvement in pass generation
Trunk dominance at release	Ratio between trunk displacement and distal arm displacement within a short release-centered window (-)	Localized indicator of trunk contribution in the final phase of force transmission

Table S14. Passing — Additional Inter-Limb Comparison Descriptors

Feature	Description (unit)	Functional interpretation
Speed difference (L−R)	Difference in peak wrist speed pre-release between left- and right-arm passes (m/s)	Inter-limb asymmetry of passing capacity
Postural-cost difference (L−R)	Difference in postural cost of passing between left- and right-arm conditions (-)	Between-side difference in the stability burden associated with passing
Release-timing difference (L−R)	Difference in release-to-peak wrist timing between left- and right-arm conditions (s)	Between-side difference in the temporal organization of force transmission
Coordination difference (L−R)	Difference in proximal–distal lag pre-release between left- and right-arm conditions (s)	Between-side difference in kinetic-chain sequencing
Release-height difference (L−R)	Difference in wrist height relative to the trunk at release between left- and right-arm conditions (m)	Between-side difference in effective release geometry

Table S15. Non-Contested Shooting — Tier 1 Postural Control Descriptors

Feature	Description (unit)	Functional meaning
Ball release time	Timestamp of automatically detected ball release (s)	Reference instant for time-locking the shooting gesture
Trunk horizontal sway at release RMS	Root mean square horizontal displacement of the trunk reference during a short release-centered window (m)	Postural stability under explosive load at the instant of ball release
Trunk vertical level variability pre-release	Standard deviation of vertical trunk position during the fixed pre-release window (m)	Ability to maintain vertical alignment while preparing the shooting action
Preparatory trunk stability	Root mean square trunk speed during the fixed pre-release window (m/s)	Ability to establish a stable posture prior to force transmission
Torso height at release	Vertical position of the trunk reference at ball release (m)	Body elevation reached at the instant of release
Post-shot recovery sway	Root mean square trunk horizontal sway during the fixed post-release window (m)	Stability recovery following high-intensity propulsion
Postural cost of shooting	Ratio between release-centered trunk horizontal sway and peak wrist speed in the pre-release window (-)	Stability sacrifice required to achieve explosive propulsion

Table S16. Non-Contested Shooting — Tier 2 Upper-Limb Explosive Capacity, Release Geometry, and Loading Descriptors

Feature	Description (unit)	Functional meaning
Peak wrist speed pre-release	Maximum magnitude of wrist velocity relative to the trunk during the fixed pre-release window (m/s)	Core indicator of residual upper-limb propulsion capacity
Peak wrist acceleration pre-release	Maximum wrist acceleration during the fixed pre-release window (m/s^2)	Explosiveness and rate of force generation
Release-to-peak wrist timing	Time difference between ball release and peak wrist speed (s)	Temporal organization of force transmission relative to release
Wrist height at release	Vertical wrist position at ball release (m)	Absolute release height
Wrist-to-torso vertical gap at release	Difference between wrist and trunk vertical positions at ball release (m)	Release geometry independent of overall body elevation
Pre-release vertical wrist lift	Net vertical displacement of the wrist from pre-release window start to release (m)	Upward arm-lift contribution during final preparation
Elbow vertical range of motion pre-release	Difference between maximum and minimum elbow vertical position relative to the trunk during the pre-release window (m)	Amplitude of upper-limb excursion contributing to propulsion
Elbow–wrist coordination pre-release	Maximum cross-correlation between elbow and wrist speed profiles during the pre-release window (-)	Integrity of proximal-distal kinetic-chain sequencing
Proximal–distal lag pre-release	Temporal delay at maximum elbow–wrist speed correlation during the pre-release window (s)	Direction and timing of intersegmental coordination
Horizontal loading excursion of wrist	Maximum backward horizontal wrist excursion relative to the release-side trajectory during the pre-release window (m)	Magnitude of a broad loading phase preceding forward propulsion
Forward wrist excursion after loading	Horizontal wrist excursion from the loading extremum to the release instant (m)	Final forward transfer of the loaded arm into release
Extra pre-release wrist path	Difference between the total effective forward excursion and the monotonic no-loading trajectory during the pre-release window (m)	Additional path generated by a distinct horizontal loading phase; higher values indicate clearer cocking behavior
Wrist loading duration	Time elapsed between the loading extremum and ball release (s)	Duration of the final horizontal loading phase before release
Wrist prerelease distance from release peak	Maximum distance between wrist position and release position during the pre-release window (m)	Overall preparatory separation from the release configuration, independent of loading direction
Wrist compact loading excursion	Maximum compact pre-release wrist excursion relative to the final release-oriented trajectory (m)	Magnitude of a short, localized preparatory phase preceding release
Wrist compact loading duration	Time elapsed between the compact loading extremum and ball release (s)	Duration of compact pre-release organization before release
Wrist loading curvature index	Curvature-based descriptor of the pre-release wrist trajectory (-)	Degree to which the preparatory path is structured rather than monotonic; higher values indicate a more evident compact loading phase

Table S17. Non-Contested Shooting — Tier 3 Compensatory Strategy and Propulsion-Origin Descriptors

Feature	Description (unit)	Functional meaning
Relative wrist propulsion index pre-release	Ratio between wrist displacement relative to the trunk and trunk displacement during the pre-release window (-)	Extent to which propulsion is arm-driven versus trunk-mediated
Torso displacement pre-release RMS	Root mean square displacement of the trunk reference during the pre-release window (m)	Degree of whole-body involvement in propulsion
Trunk rotation variability pre-release	Standard deviation of trunk rotation proxy angle during the pre-release window (deg)	Rotational compensation strategy during force generation
Shoulder asymmetry at release	Absolute vertical difference between right and left shoulders at ball release (m)	Asymmetric upper-body loading or compensatory elevation
Pre-release vertical trunk lift	Net vertical displacement of the trunk reference from pre-release window start to release (m)	Buoyancy-assisted or trunk-driven elevation contributing to propulsion
Trunk dominance at release	Ratio between trunk displacement and distal arm displacement within a short release-centered window (-)	Localized indicator of trunk contribution to propulsion at the instant of ball release; higher values indicate greater trunk involvement in the final phase of force transmission

Table S18. Contested Shooting under Physical Opposition — Tier 1 Resistance and Recovery Descriptors

Feature	Description (unit)	Functional interpretation
Submersion time	Time interval between the last valid pre-contact frame and first valid re-emergence frame (s)	Ability to tolerate externally imposed perturbation and recover upright posture
Re-emergence success	Binary indicator of successful above-water reappearance after contact (-)	Fundamental recovery capacity under contact
Post-contact preparation time to release, attempted release	Time between re-emergence and ball release, or attempted release, in Phase 3 (s)	Efficiency of motor reorganization after perturbation before functional completion
Ball release success	Binary indicator of successful ball release after contact (-)	Ability to translate recovery into a functional shooting action
Shot completion level	Ordinal descriptor: complete release / partial release / no release (-)	Graded functional outcome under contested conditions
Action outcome category	Categorical descriptor summarizing trial outcome (completed, delayed, partial, aborted) (-)	Global functional performance under physical opposition

Table S19. Contested Shooting under Physical Opposition — Tier 2 Post-Contact Motor Reorganization Descriptors

Feature	Description (unit)	Functional interpretation
Reorganization window duration	Duration of the valid contiguous Phase 3 segment preceding ball release or trial termination (s)	Stability and motor control regained after perturbation
Trunk stabilization variability pre-release	RMS horizontal displacement of the trunk reference during the fixed pre-release window in Phase 3 (m)	Ability to rapidly re-establish postural control before release
Vertical trunk recovery amplitude	Net vertical displacement of the trunk reference from re-emergence to release (m)	Active buoyancy recovery and vertical repositioning capacity
Early trunk rotational variability	Standard deviation of trunk rotation proxy angle during early Phase 3 (deg)	Compensatory rotational adjustments after contact
Reorganization smoothness	RMS jerk of the trunk reference during early Phase 3 (m/s^3)	Quality of recovery control; higher values indicate fragmented re-stabilization
Torso height at contested release	Vertical position of the trunk reference at ball release in Phase 3 (m)	Body elevation recovered before action completion

Table S20. Contested Shooting under Physical Opposition — Tier 3 Post-Contact Shooting Descriptors

Feature	Description (unit)	Functional interpretation
Peak wrist speed pre-release	Maximum wrist speed relative to the trunk during the fixed pre-release window in Phase 3 (m/s)	Residual upper-limb propulsion capacity after perturbation
Release-to-peak wrist timing	Time difference between ball release and peak wrist speed in Phase 3 (s)	Explosiveness and temporal organization of force generation following contact
Proximal–distal lag pre-release	Temporal delay at maximum elbow–wrist speed correlation during the pre-release window in Phase 3 (s)	Robustness of intersegmental coordination under pressure
Relative wrist propulsion index pre-release	Ratio between wrist displacement relative to the trunk and trunk displacement during the pre-release window in Phase 3 (-)	Shift toward trunk-driven propulsion after contact
Wrist height at contested release	Vertical wrist position at ball release in Phase 3 (m)	Effective release height under perturbation
Trunk dominance at release	Ratio between trunk displacement and distal arm displacement within a short release-centered window around contested release (-)	Compensatory whole-body involvement required to complete the action

Table S21. Contested Shooting under Physical Opposition — Tier 4 Derived Integrated Descriptors

Feature	Description	Functional interpretation
Recovery Efficiency Index	Peak wrist speed pre-release divided by the sum of submersion time and post-contact preparation time to release (m/s^2)	Efficiency in converting post-contact recovery into effective propulsion
Compensation Burden Index	Trunk dominance at release multiplied by the sum of submersion time and post-contact preparation time to release (s)	Overall compensatory burden required to recover and complete the action

Table S22. Descriptor set for within-subject, within-arm comparison between non-contested shooting and post-contact contested shooting

Feature	Description (unit)	Functional interpretation
Peak wrist speed pre-release	Difference in peak wrist speed between non-contested and contested post-contact shooting (m/s)	Change in distal propulsion capacity after perturbation relative to baseline shooting
Release Height Retention Ratio	Ratio between contested and non-contested release height relative to the trunk (-)	Extent to which release geometry is preserved or altered after perturbation relative to baseline shooting
Ratio peak wrist speed pre-release	Contested / non-contested peak wrist speed pre-release (-)	Relative preservation of propulsion capacity
Release-to-peak timing	Difference in release-to-peak wrist timing between conditions (s)	Change in the temporal organization of force transmission relative to release after perturbation
Proximal–distal lag pre-release	Difference in proximal–distal lag between conditions (s)	Change in intersegmental sequencing after perturbation relative to baseline shooting
Relative wrist propulsion index pre-release	Difference or ratio in relative wrist propulsion index between conditions (-)	Change in the balance between arm-driven propulsion and trunk-mediated contribution under perturbation
Wrist height at release	Difference in wrist height at release between conditions (m)	Change in release geometry under perturbation
Torso height at release	Difference in torso height at release between conditions (m)	Change in body elevation achieved at release under perturbation
Trunk dominance at release	Difference in trunk dominance at release between conditions (-)	Change in compensatory whole-body contribution during the final phase of force transmission under perturbation

General note. These descriptors are intended to characterize how shooting execution is preserved, modified, or reorganized after perturbation relative to the non-contested condition. Accordingly, they should not be interpreted as pure degradation scores, since contested performance may reflect a mixture of reduced output, preserved release organization, or compensatory reorganization depending on the subject and arm.
